# Surface Functionalization of Surfactant‐Free Particles: A Strategy to Tailor the Properties of Nanocomposites for Enhanced Thermoelectric Performance

**DOI:** 10.1002/anie.202207002

**Published:** 2022-07-21

**Authors:** Cheng Chang, Yu Liu, Seung Ho Lee, Maria Chiara Spadaro, Kristopher M. Koskela, Tobias Kleinhanns, Tommaso Costanzo, Jordi Arbiol, Richard L. Brutchey, Maria Ibáñez

**Affiliations:** ^1^ Institute of Science and Technology Austria Am Campus 1 3400 Klosterneuburg Austria; ^2^ Catalan Institute of Nanoscience and Nanotechnology (ICN2) CSIC, and BIST 08193 Barcelona, Catalonia Spain; ^3^ ICREA Pg. Lluís Companys 23 08010 Barcelona, Catalonia Spain; ^4^ Department of Chemistry University of Southern California Los Angeles CA 90089 USA

**Keywords:** Thermoelectrics, SnTe, Grain Boundary, Alkahest Solutions, Surface Functionalization

## Abstract

The broad implementation of thermoelectricity requires high‐performance and low‐cost materials. One possibility is employing surfactant‐free solution synthesis to produce nanopowders. We propose the strategy of functionalizing “naked” particles’ surface by inorganic molecules to control the nanostructure and, consequently, thermoelectric performance. In particular, we use bismuth thiolates to functionalize surfactant‐free SnTe particles’ surfaces. Upon thermal processing, bismuth thiolates decomposition renders SnTe‐Bi_2_S_3_ nanocomposites with synergistic functions: 1) carrier concentration optimization by Bi doping; 2) Seebeck coefficient enhancement and bipolar effect suppression by energy filtering; and 3) lattice thermal conductivity reduction by small grain domains, grain boundaries and nanostructuration. Overall, the SnTe‐Bi_2_S_3_ nanocomposites exhibit peak *z* *T* up to 1.3 at 873 K and an average *z* *T* of ≈0.6 at 300–873 K, which is among the highest reported for solution‐processed SnTe.

## Graphical abstract

A novel strategy of functionalizing the surface of “naked” particles by using inorganic molecules is proposed. In particular, bismuth thiolate were used to functionalize surfactant‐free SnTe particles’ surfaces. Upon thermal processing, bismuth thiolates decomposition renders SnTe‐Bi_2_S_3_ nanocomposites with synergistic functions to enhance thermoelectric performance.

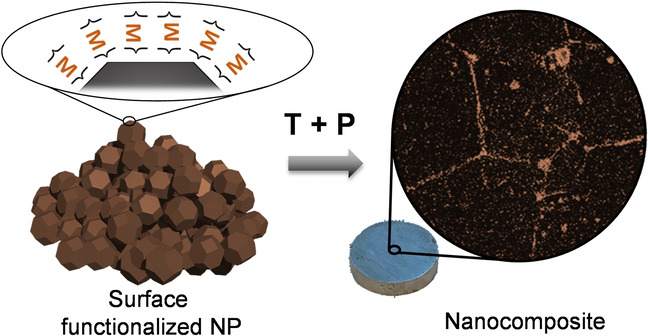

## Introduction

Thermoelectric materials directly and reversibly transform heat and electricity. Considering heat as the most common form of waste energy, this phenomenon has been explored as a potential source of clean, non‐fossil energy.^[1]^ The conversion efficiency of thermoelectric materials is determined by the dimensionless figure of merit, *z* *T*=*S* ^2^ *σ* *T*/*κ*, where *S*, *σ*, *κ*, and *T* are the Seebeck coefficient, electrical conductivity, thermal conductivity, and absolute temperature, respectively. During the last century, material scientists have tried to maximize materials’ *z* *T* to enhance conversion efficiency. However, the complex and predominantly counterproductive relationships between the parameters determining *z* *T* make it challenging.^[2]^

The most successful strategies to enhance performance have been based on refining the materials’ band structures and scattering centers simultaneously, which require superb control over the material structural properties.^[3]^ Defects ranging from atomic impurities to embedded nanoparticles must be precisely engineered to maximize electron transport while hampering phonon movement. Inorganic nanocomposites are among the most suitable materials to tackle such endeavors. There are plenty of methods to produce inorganic nanocomposites, the most common in thermoelectric materials being spinodal decomposition in high‐temperature reactions. This synthetic strategy delivered record *z* *T*s in different material systems, e.g. PbTe, SnTe, or SiGe.^[4]^ While successful, it requires large amounts of energy, long reaction time, and ultra‐high purity reagents, therefore, yielding materials that are too expensive for large scale implementation.

Alternatively, in the last decade, solution processing methods have been explored to reduce material production costs. Solution syntheses commonly use low‐cost reagents, low temperatures, and short reaction time to produce powders. The most widely used method to produce powders in solution for thermoelectric materials is the so‐called surfactant‐free since it has high reaction yields and renders particles without insulating long‐chain organic molecules, typically found when surfactants are used.^[5]^

From particles to dense materials, sintering is necessary.^[6]^ The sintering process involves densification and particle coarsening, both of which will determine the microstructure of the nanocomposite.^[7]^ Particle surfaces play a crucial role in all these phenomena and therefore modifying their characteristics is a powerful strategy to engineer nanocomposite structural properties.

One possibility, barely explored, is the particle surface functionalization with different organic or inorganic molecules. However, the high temperatures employed, usually above 400 °C, will decompose those surface species. While this can be seen as a hurdle, it also represents an opportunity. The decomposition products can be used to control the matrix composition or even to form different types of secondary phases. Herein, we demonstrate that adding surface molecules to “naked” particles and tailoring their decomposition is a powerful tool for precisely controlling nanocomposite structural and thermoelectric properties in SnTe. In particular, we prepare bismuth thiolates in a thiol‐amine mixture. These molecular solutes are then used to functionalize surfactant‐free SnTe particles’ surfaces. The surface‐modified SnTe particles are then subjected to various thermal processes that yield dense SnTe‐Bi_2_S_3_ nanocomposites. Depending on the precursors used to prepare the bismuth thiolates, different decomposition products are obtained, resulting in nanocomposites with distinct structural properties. The produced SnTe‐Bi_2_S_3_ nanocomposites exhibit peak *z* *T* up to 1.3 at 873 K and an average *z* *T* of ≈0.6 at 300–873 K, which is among the highest reported for SnTe.

## Results and Discussion

Ligand‐free SnTe nanoparticles (NPs) were prepared in water at ambient pressure using SnCl_2_ and Te as precursors. This synthetic method was chosen due to its simplicity and uses very inexpensive precursors. The resulting SnTe NPs have an irregular shape with dimensions of 20–50 nm (Figure S1). The as‐synthesized SnTe NPs were purified from the reaction byproducts by precipitation, rinsing with ethanol and acetone. The washed NPs were then dried under vacuum overnight at room temperature, annealed in forming gas (95 % N_2_+5 % H_2_), and consolidated into pellets under vacuum using the spark plasma sintering technique. Further details of the entire process can be found in the Supporting Information.

**Figure 1:**
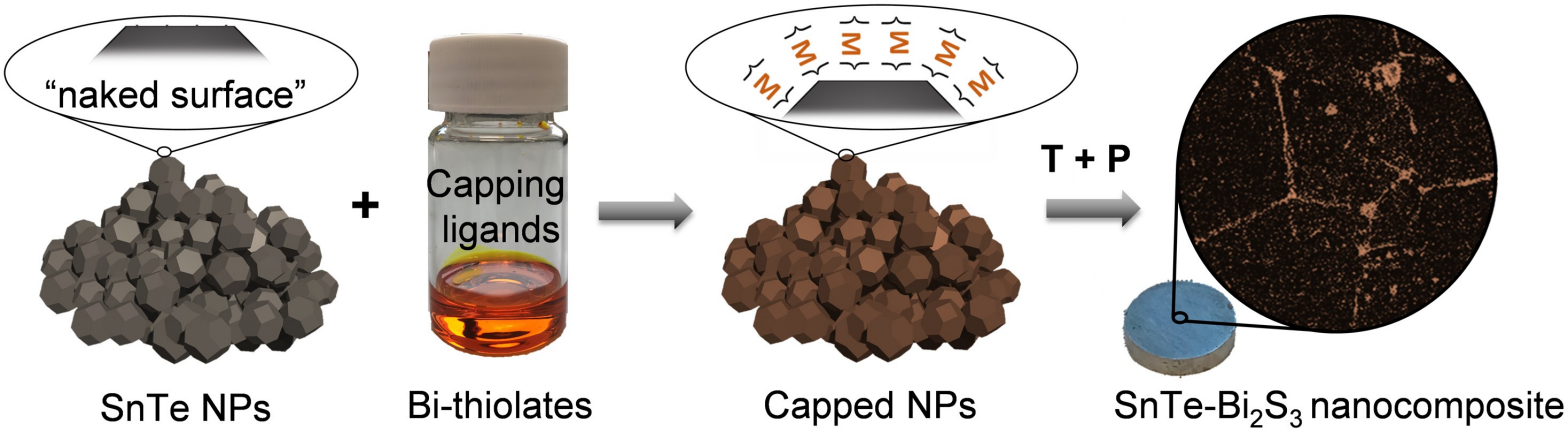
SnTe‐Bi_2_S_3_ nanocomposite synthetic process: SnTe NPs are capped with bismuth thiolates, then washed, dried, annealed, and consolidated into a pellet by applying temperature (T) and pressure (P), forming the SnTe‐Bi_2_S_3_ nanocomposites.

To form SnTe‐Bi_2_S_3_ nanocomposites, SnTe NPs were dispersed in *N*‐methyl formamide, then different amounts (*x* mol %) of dissolved Bi_2_O_3_ or Bi_2_S_3_ in a thiol‐amine solution (1,2‐ethanedithiol, EDT; ethylenediamine, en) were added. The mixtures were vigorously stirred at room temperature in an N_2_ filled glovebox for 24 h. The treated SnTe NPs were then precipitated from the solution, washed with acetone thoroughly, and dried under vacuum for further processing into pellets following the same steps as for the bare SnTe NPs, resulting in dense SnTe‐Bi_2_S_3_ nanocomposites (Figure 1). The nanocomposites produced with Bi_2_O_3_ solutions are referred to as SnTe‐*x* %Bi_2_S_3_, where *x*=0.5, 1, 1.5, 2, 2.5, and 3. The nanocomposites produced with Bi_2_S_3_ solutions are referred to as SnTe‐*x* %*Bi_2_S_3_*, where *x*=1, 2, and 3.

**Figure 2:**
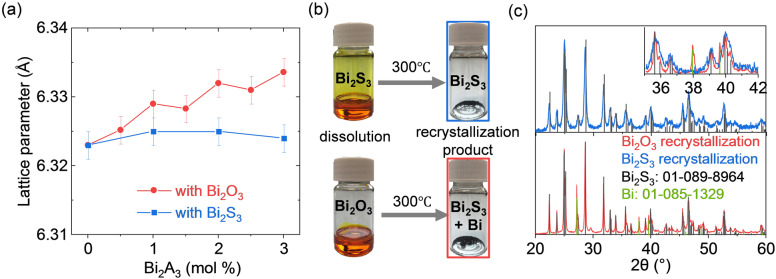
a) The SnTe lattice parameter of SnTe‐Bi_2_S_3_ nanocomposite produced with Bi_2_O_3_/Bi_2_S_3_ solutions. b) Pictures of the Bi_2_O_3_/Bi_2_S_3_ solutions and the resulting products after annealing at 300 °C under vacuum. c) The XRD patterns of recrystallization products of Bi_2_O_3_/Bi_2_S_3_ solutions.

The X‐ray diffraction (XRD) patterns of the consolidated SnTe‐Bi_2_S_3_ nanocomposites produced with the particles treated with Bi_2_O_3_ or Bi_2_S_3_ solutions match the single phase of the SnTe rock‐salt structure without any visible crystalline impurity phases detected (Figure S2). In the case of treating SnTe NPs with the Bi_2_O_3_ solution, the lattice parameter of SnTe increased with the Bi_2_O_3_ molar content used to treat the SnTe NPs (Figure 2a). On the contrary, the SnTe lattice parameters of the nanocomposites prepared with Bi_2_S_3_ solution remain almost unchanged despite increasing the Bi_2_S_3_ content. To understand the differences between the composites prepared using Bi_2_O_3_ and Bi_2_S_3_, we looked at the identity of the molecular solutes for solutions of Bi_2_O_3_ and Bi_2_S_3_ dissolved in 1 : 10 (vol/vol) mixtures of EDT and en at room temperature and ambient pressure. In particular, we performed direct‐injection electrospray ionization mass spectrometry (ESI‐MS) using a liquid chromatography quadrupole time‐of‐flight mass spectrometer (LC/Q‐TOF‐MS) and UV/Vis spectroscopy measurements. Figure S3 shows the negative ion mode mass spectra for Bi_2_O_3_ and Bi_2_S_3_ solutions, respectively. The three most abundant peaks at *m*/*z*=272.9, 332.9, and 392.9 match well with the calculated values for [BiS_2_]^−^, [BiS(C_2_H_4_S_2_)]^−^ (or BiS(EDT)^−^), and [Bi(C_2_H_4_S_2_)_2_]^−^ (or Bi(EDT)_2_^−^), respectively, for both samples with the other peaks matching to those of the solvent blank (Table 1). Positive‐ion mode mass spectra peaks most likely correspond to various alkylammonium species as reported previously for other thiol‐amine solutions.^[8]^ UV/Vis absorption spectroscopy was used to further investigate the absorption signatures of these molecular solutes. Dilute solutions of both Bi_2_O_3_ and Bi_2_S_3_ exhibit an absorption band at *λ*_max_=392 nm (Figure S4) that is assigned to a ligand‐to‐metal charge transfer band (LMCT), consistent with other experimentally observed and computationally predicted bismuth(III) thiolate complexes.^[9]^ This suggests that both Bi_2_O_3_ and Bi_2_S_3_ yield similar bismuth thiolate solutes upon dissolution, corroborating the conclusions drawn from ESI‐MS measurements.

**Table 1:** Molecular formulas and proposed structures for ions observed in negative‐ion mode mass spectra.

Measured *m*/*z*	Molecular formula	Proposed structures	Calculated *m*/*z*
272.9	[BiS_2_]^−^	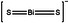	273.1
332.9	[BiS(C_2_H_4_S_2_)]^−^	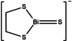	333.2
392.9	[Bi(C_2_H_4_S_2_)_2_]^−^	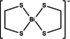	393.3

While indirect characterization methods such as those used here (ESI‐MS, UV/Vis) point to mostly identical solutes resulting from the two precursors, we found distinct differences after recrystallizing them by annealing under vacuum at 300 °C. The XRD patterns reveal that Bi_2_O_3_ solution transforms into a biphasic Bi_2_S_3_ and metallic Bi mixture, whereas the Bi_2_S_3_ solution yields phase‐pure Bi_2_S_3_ (Figures 2b, c).^[10]^ We hypothesize that the differences during crystallization of both solutions comes from the evolution of stoichiometric amounts of H_2_O from the liberation of lattice oxygen in Bi_2_O_3_, as seen in previous studies.^[11]^

(1) $$e.g.\;Bi_{2}O_{3}+4\;C_{2}H_{6}S_{2}\to 2\;{\left[Bi{\left(C_{2}H_{4}S_{2}\right)}_{2}\right]}^{-}+2\;H^{+}+3\;H_{2}O$$

Since both Bi_2_O_3_ and Bi_2_S_3_ solutions are constituted of the same molecular species (Table 1), we attributed the variation in SnTe lattice parameter to the differences seen in the decomposition products of the two solutions. During the thermal processing in the nanocomposite produced with the Bi_2_O_3_ solution, we hypothesize that Bi produced during the decomposition diffuses into SnTe lattice occupying Sn or Sn vacancy sites, leading to the lattice expansion. Thus, Bi^III^ should also act as an aliovalent dopant modifying the charge carrier concentration, as is confirmed later on.

### Nanocomposite Structural Properties

**Figure 3:**
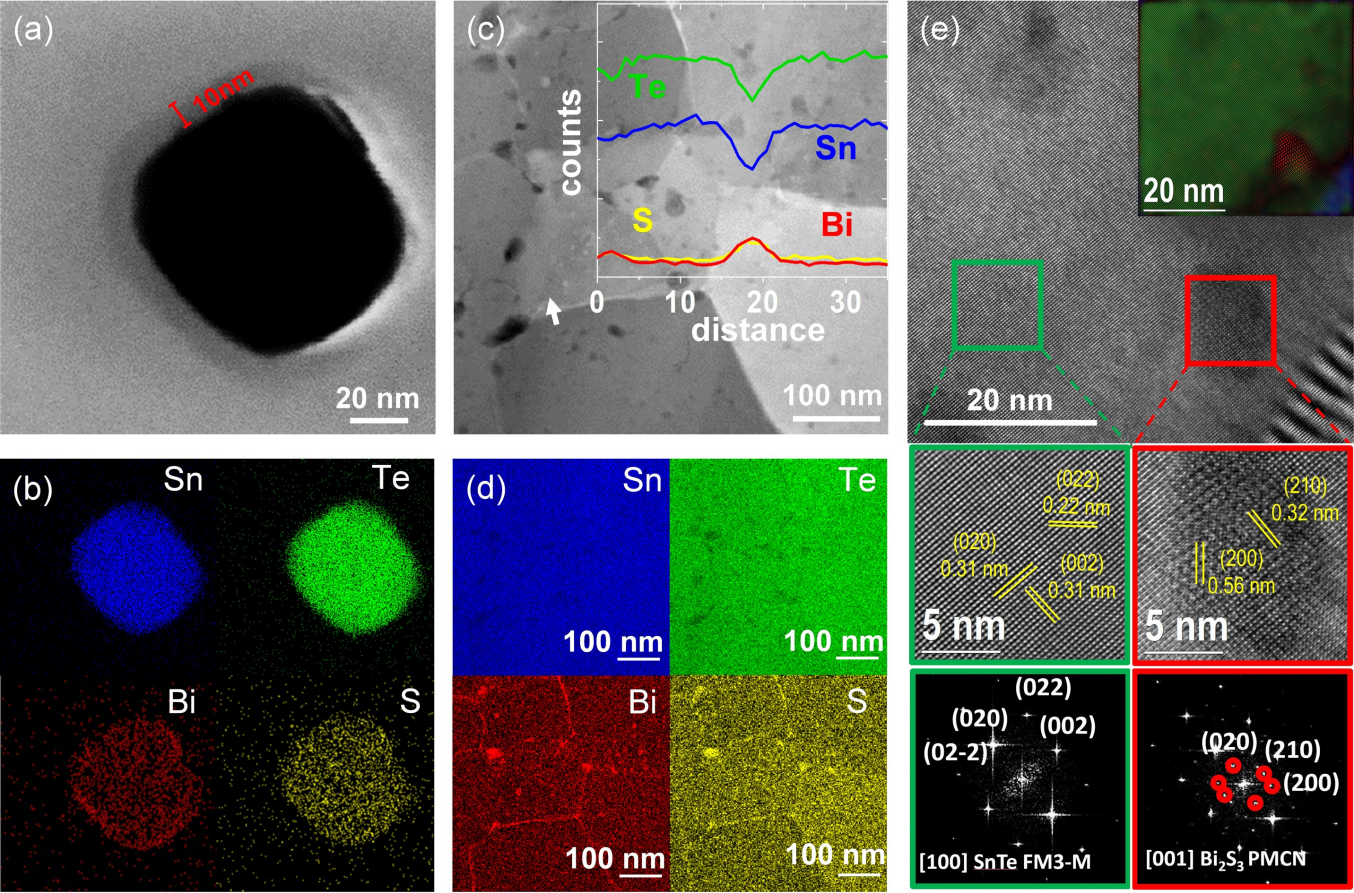
a) STEM image of a SnTe NP coated with bismuth thiolate complexes from the dissolution of Bi_2_O_3_ in the alkahest solvent, and b) its corresponding EDS maps. c) STEM of SnTe‐1.5 %Bi_2_S_3_ nanocomposite produced with the Bi_2_O_3_ solution. The inset image is the linear EDS spectrum along the white arrow direction. d) The corresponding EDS maps. e) HRTEM image of SnTe‐1.5 %Bi_2_S_3_ nanocomposite produced with Bi_2_O_3_ solution. Inset, the phase‐filtered structural map shows the SnTe phase marked as green and blue with different orientations and Bi_2_S_3_ marked as red. The inset top right shows the crystallographic mapping of two adjacent SnTe grains with a Bi_2_S_3_ nanoprecipitate in between.

Transmission electron microscopy (TEM) related analysis of the surface‐treated NPs showed a ca. 10 nm amorphous shell around the SnTe NPs constituted of homogeneously distributed Bi and S atoms (Figures 3a, b). We attribute this amorphous shell to the adsorption of the different bismuth thiolates: [BiS_2_]^−^, [BiS(C_2_H_4_S_2_)]^−^ and [Bi(C_2_H_4_S_2_)_2_]^−^.

To evaluate the microstructure of the nanocomposites, the SnTe‐1.5 %Bi_2_S_3_ pellet was thinned to electron transparency by Ar+ polishing to produce a self‐suspended lamella. Figure 3c shows the linear energy‐dispersive X‐ray spectrometry (EDS) scan along the selected grain boundary (white arrow). Quantitative results reveal evident Sn/Te spectrum valleys and Bi/S spectrum peaks at the grain boundary areas, indicating that during the thermal processing, the bismuth complexes crystallize, forming Bi_2_S_3_ secondary phases because of the low solubility of Bi into SnTe.^[12]^ Compositional maps show that Bi_2_S_3_ is mostly located along the grain boundaries forming grain boundary complexions and in the form of Bi_2_S_3_ nanodots (Figure 3d). High‐resolution transmission electron microscopy (HRTEM) verified the presence of Bi_2_S_3_ nanoprecipitates with an orthorhombic crystal structure between SnTe crystalline domains (Figure 3e). In particular, the marked red region corresponds to the Bi_2_S_3_ visualized along the [001] zone axis and the selected green region corresponds to the SnTe visualized along the [100] zone axis parallel to the electron beam direction. In the inset of Figure 3e, the corresponding phase‐filtered structural mapping is shown, where the SnTe phase from two adjacent grains is marked as green and blue, and the Bi_2_S_3_ phase is displayed in red. The Bi_2_S_3_ phase lies between the two misoriented adjacent SnTe grains, consistent with the EDS results. Additional STEM, SEM, EDS, and HRTEM data can be found in Figures S5–S15 and Table S1.

**Figure 4:**
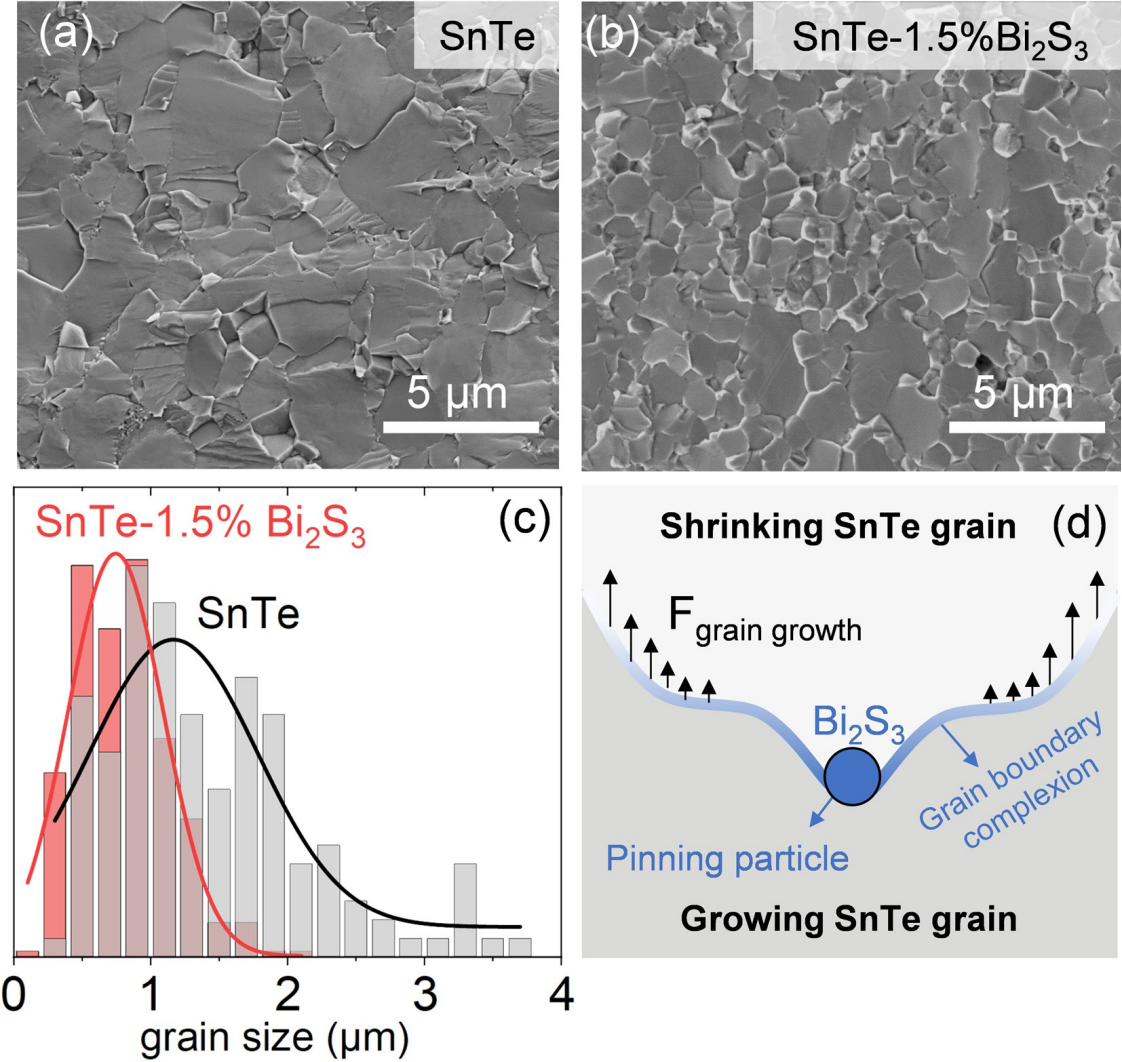
SEM image of a) bare SnTe, b) SnTe‐1.5 %Bi_2_S_3_ nanocomposite produced with the Bi_2_O_3_ solution. c) Grain size distribution and corresponding Gaussian distribution fitting, and d) schematic of grain growth inhibition in the SnTe‐Bi_2_S_3_ nanocomposite.

Figure 4 compares the scanning electron microscopy images of the pellets produced with bare SnTe NPs and those treated with 1.5 % Bi_2_O_3_ solution. SEM images of the other SnTe‐*x* %Bi_2_S_3_ nanocomposites formed with different amounts of Bi_2_O_3_ solution can be found in Figure S8. SnTe‐1.5 %Bi_2_S_3_ nanocomposite contains smaller grains than bare SnTe. The average grain size decreases from 1.17 μm in bare SnTe to 0.74 μm in SnTe‐1.5 %Bi_2_S_3_ nanocomposite, and the variance also reduces from 1.23 to 0.7 μm (Figure 4c). We associate these with the presence of Bi_2_S_3_ secondary phase as nanoprecipitates at the grain boundaries.

Grains grow through atomic diffusion across the grain boundaries, which can be envisioned as grain boundary movement.^[13]^ The presence of Bi_2_S_3_ at the grain boundary inhibits grain growth due to the stagnant diffusion of SnTe through Bi_2_S_3_. Moreover, Bi_2_S_3_ nanoprecipitates act as pinning centers hindering grain boundary movement, a phenomenon known as Zenner pinning (Figure 4d).^[14]^ As a result, mass diffusion is kinetically stabilized, hindering grain growth. The same effect can be observed in SnTe‐Bi_2_S_3_ nanocomposites formed with the Bi_2_S_3_ solution (Figure S9).

### Thermoelectric Properties

**Figure 5:**
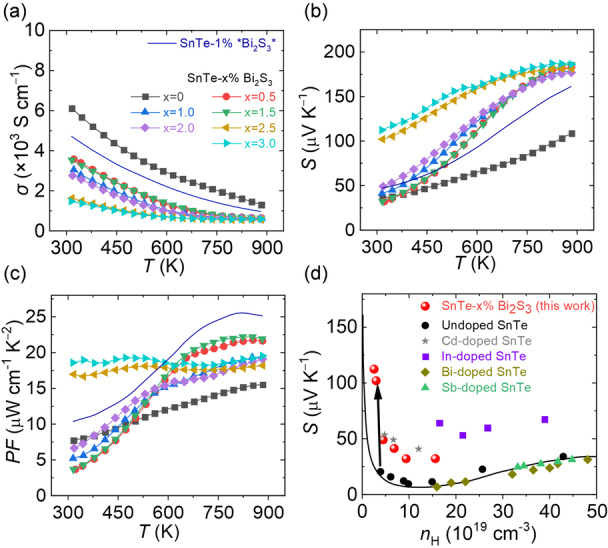
The electrical transport properties of SnTe‐*x* %Bi_2_S_3_ nanocomposites produced with the Bi_2_O_3_ solution. a) Electrical conductivity. b) Seebeck coefficient. c) Power factor. d) The Pisarenko plot of SnTe according to the two‐band Kane model in reference [17]. The Seebeck coefficient of undoped/Bi‐/Sb‐/In‐/Cd‐ doped SnTe using the traditional melting method are taken from reference [16].

All samples exhibit heavily doped semiconductor behavior, with *σ* decreasing with rising temperatures (Figure 5a). SnTe is intrinsically p‐type due to its off‐stoichiometric nature with a large number of Sn lattice vacancies that yields very high intrinsic doping levels.^[15]^ Noticeably, the room temperature *σ* decreases from ≈6100 S cm^−1^ for bare SnTe to ≈3500 S cm^−1^ for SnTe‐3 %Bi_2_S_3_ nanocomposites with the carrier concentration (*p*_H_) reduces from 1.4×10^21^ cm^−3^ to 3.0×10^19^ cm^−3^. Such electrical conductivity and carrier concentration changes are associated with Bi‐doping, which acts as an electron donor, reducing the overall hole concentration. The nanocomposite prepared with 1 mol % Bi_2_S_3_ solution referred to as SnTe‐1 %*Bi_2_S_3_* is also presented for comparison. As expected, *σ* in SnTe‐1 %*Bi_2_S_3_* is much higher than SnTe‐1 % Bi_2_S_3_ due to the absence of Bi doping evidenced by the lattice parameters. The data corresponding to all the SnTe‐*x* %*Bi_2_S_3_* nanocomposites can be found in Figures S16.

Bare SnTe and all the produced SnTe‐*x* %Bi_2_S_3_ nanocomposites have a positive *S*, which increases with temperature (Figure 5b). *S* values of all SnTe‐Bi_2_S_3_ nanocomposites approach the same maximum value of ≈180 μV K^−1^ at 873 K. The room‐temperature *S* increases with *x* from ≈35 μV K^−1^ for bare SnTe to ≈110 μV K^−1^ for SnTe‐3 %Bi_2_S_3_ nanocomposite. The combination of the optimized *σ* and enhanced *S* leads to high power factors (*PF*=*σS* ^*2*^) in SnTe‐Bi_2_S_3_ nanocomposites (Figure 5c). Surprisingly, the composites with 2.5 % and 3 %Bi_2_S_3_ exhibit a steady high *PF* within the whole temperature range studied, mainly deriving from the high *S* at low temperatures. The same phenomenon happens for SnTe‐3 %*Bi_2_S_3_* as well.

To get an insight into the high *S*, we investigated the room‐temperature *S* of SnTe‐Bi_2_S_3_ nanocomposites as a function of *p*_H_, and compared it with bare, Bi‐, Sb‐, Cd‐, and In‐doped SnTe from previous works (Figure 5d).^[16]^ The theoretical Pisarenko relationship was calculated based on a two‐valence‐band model.^[17]^ *S* values of Cd‐ and In‐doped SnTe lie above the calculated Pisarenko line due to the band convergence effect and resonant doping, respectively.^[18]^ On the other hand, *S* values of the previously reported Bi‐and Sb‐doped SnTe fit well with the estimated Pisarenko, indicating Bi and Sb make no noticeable difference in the SnTe band structure.^[19]^ However, in this work, all *S* values of SnTe‐Bi_2_S_3_ nanocomposites lie far above the Pisarenko line. We attribute this to the energy filtering occurring due to the presence of Bi_2_S_3_ rich grain boundaries found in the TEM study. Figure S17 illustrates the relative band structure energy of SnTe and Bi_2_S_3_ to vacuum.^[20]^ The discrepant band structures between SnTe and Bi_2_S_3_ establish effective potential barriers in which low‐energy charge carriers are filtered out. In contrast, high‐energy charge carriers can pass through the barrier freely, leading to higher DOS effective mass (m*). We calculated m* of all SnTe‐Bi_2_S_3_ nanocomposites, according to m*≈*m*_e_(*μ*_w_/*μ*_H_)^2/3^ (Hall mobility *μ*_H_, weighted mobility *μ*_w_ and m* can be found in Table S2 and Figure S18),^[21]^ confirming that m* in SnTe‐Bi_2_S_3_ nanocomposites is higher than that of bare SnTe. Apart from the energy filtering effect, the potential barrier is also responsible for the carrier concentration reduction since low energy charge carriers are trapped in the potential well, which is confirmed in SnTe‐*Bi_2_S_3_* nanocomposites where carrier concentration still decrease without Bi doping (Table S2).^[22]^

**Figure 6:**
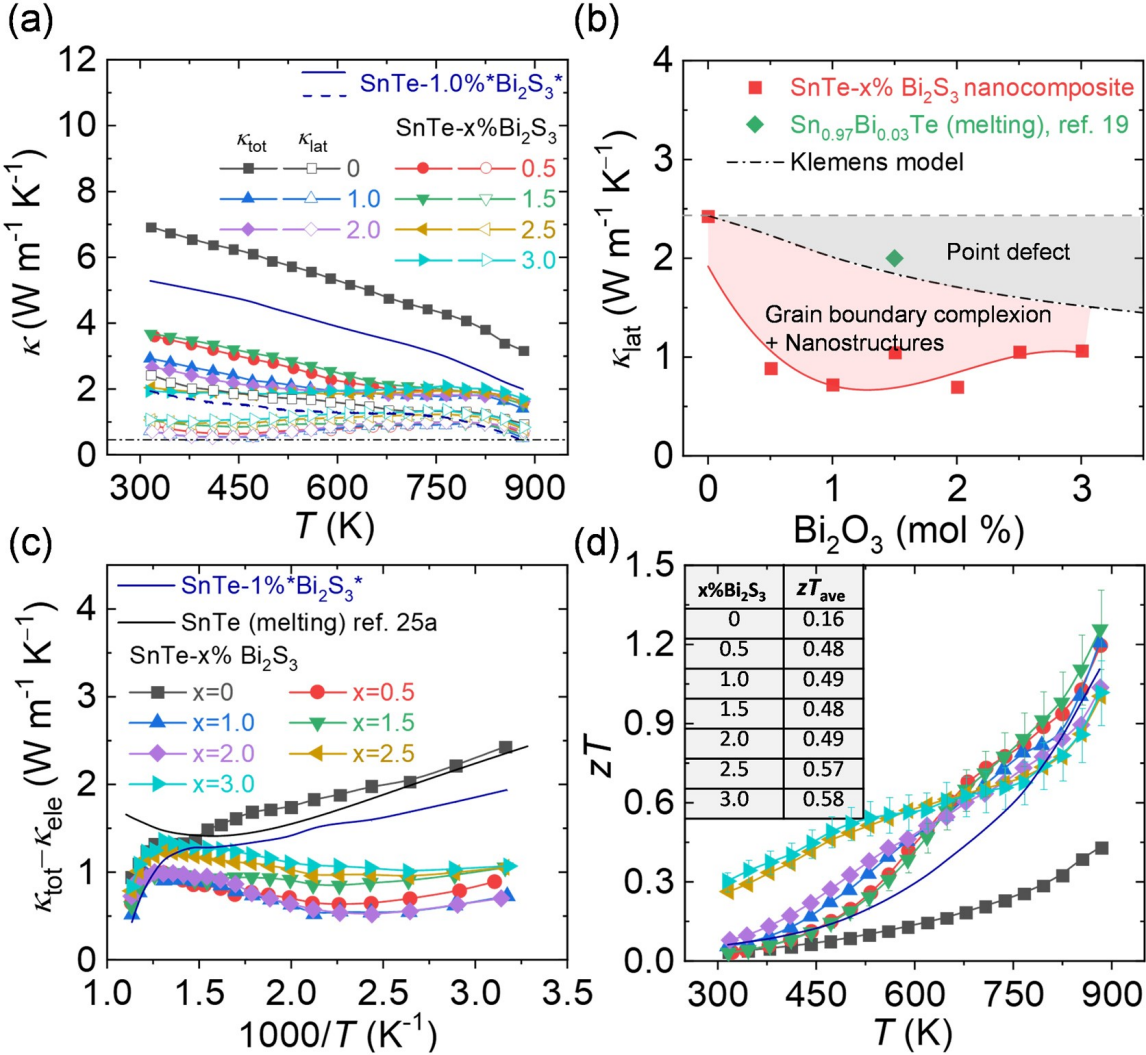
a) The total thermal conductivity (solid symbol) and lattice thermal conductivity (hollow symbol) of SnTe‐*x* %Bi_2_S_3_ nanocomposites produced with the Bi_2_O_3_ solution and SnTe‐1 %*Bi_2_S_3_* prepared with the Bi_2_S_3_ solution. b) The lattice thermal conductivity as a function of Bi_2_S_3_ amount for the SnTe‐*x* %Bi_2_S_3_ nanocomposites. The dashed black lines represent the Klemens model. c) The (*κ*_tot_−*κ*_ele_) as a function of 1000/*T*. d) The thermoelectric figure of merit *z* *T*. The inset table reveals the average *z* *T* at 300–873 K.

Aside from the enlarged *PF*, the surface functionalization with bismuth thiolates also provides effective means to reduce *κ*. Figure 6a shows the total thermal conductivity (*κ*_tot_) and lattice thermal conductivity (*κ*_lat_) as a function of temperature (specific heat *C*_p_, thermal diffusivity *α*, Lorentz number *L*, sample density *ρ* and electronic thermal conductivity *κ*_ele_ can be found in Figure S19, and Table S3. *L* is calculated using the single parabolic band (SPB) model with acoustic phonon scattering, which is also discussed in Supporting Information). The room‐temperature *κ*_tot_ of bare SnTe is ≈7 W m^−1^ K^−1^, arising from the undesirable high *κ*_ele_. Using Bi_2_O_3_ solutions can significantly suppress the high *κ*_ele_ by reducing *p*_H_ (Table S2) and introduce additional scattering sources.

We estimated *κ*_lat_ according to the Wiedemann–Franz relationship (*κ*_lat_=*κ*_tot_−*LσT*) (Figure 6a). *κ*_lat_ decreases from ≈2.5 W m^−1^ K^−1^ for bare SnTe to ≈1 W m^−1^ K^−1^ for SnTe‐Bi_2_S_3_ nanocomposites at room temperature and decreases with rising temperatures, approaching the amorphous limit at 973 K.^[23]^

We carried out Klemens model simulations to evaluate the origin of the low *κ*_lat_ in SnTe‐Bi_2_S_3_ nanocomposites (Detailed calculations can be found in Supporting Information).^[24]^ Figure 6b shows the calculated room‐temperature *κ*_lat_ of SnTe‐*x* %Bi_2_S_3_ nanocomposites and the simulated by the Klemens model as a function of *x*. As for the Klemens model, *κ*_lat_ reveals an apparent decrease due to the point defects introduced by Bi_2_S_3_, assuming Bi_2_S_3_ is fully alloyed with SnTe. However, the experimental *κ*_lat_ values in SnTe‐Bi_2_S_3_ nanocomposites are much lower than the calculated values, indicating other potential phonon scattering sources. We attribute the significant discrepancy between experimental and predicted values to the combined phonon scattering at the grain boundaries, Bi_2_S_3_ rich grain boundary complexion, and Bi_2_S_3_ nanoprecipitates. These accumulated defects, ranging from nanometers to micrometers, scatter phonons with corresponding mean free paths effectively. Together with the phonon scattering at the atomic scale, the surface treatment allows producing nanocomposites with scattering features at all length scales, which is well‐known as all‐scale hierarchical phonon scattering.^[3d]^

SnTe is a narrow band gap semiconductor; hence bipolar conduction has a detrimental effect on the thermal transport.^[25]^ The rise of *κ*_tot_ and *κ*_lat_ at ≈673 K in bare SnTe prepared by the melting method are indicative of the bipolar effect onset.^[25a]^ However, *κ* values of all SnTe‐Bi_2_S_3_ nanocomposites decrease monotonically in the whole temperature range (Figure 6c), suggesting the suppressed bipolar thermal conductivity. To further illustrate the potential bipolar effect on *κ*, we investigated *κ*_tot_−*κ*_ele_ as a function of temperature. Without the bipolar effect, *κ*_lat_ should equal to *κ*_tot_−*κ*_ele_, values and *κ*_lat_ and *T* ^−1^ should satisfy a linear relationship according to the acoustic phonon scattering assumption. Otherwise, *κ*_tot_−*κ*_ele_ curve will exhibit an upturn at the low 1000/*T* region (high temperatures), same as bare SnTe synthesized by melting method shown with a continuous black line.^[25a]^ Notably, *κ*_tot_−*κ*_ele_ of bare SnTe and SnTe‐Bi_2_S_3_ nanocomposites in this work continue to decrease upon 673 K (1.48 K^−1^ in Figure 6c), indicating the suppressed bipolar effect. We attributed this to the potential barriers at the SnTe‐Bi_2_S_3_ interface, which hinder the thermal‐activated electron transport in the conduction band at high temperatures.^[26]^

Collectively, the combination of high *PF* and low *κ* leads to a high maximum *z* *T* value of ≈1.3 at 873 K, Figure 6d, outperforming any other SnTe nanocomposites synthesized by the bottom‐up assembly, yet using a simple, scalable and cheap aqueous synthetic method. Also, room‐temperature *z* *T* is significantly improved from ≈0.04 in bare SnTe to ≈0.3 in SnTe‐3 %Bi_2_S_3_ nanocomposite, leading to a remarkable average *z* *T* upgrade in 300–873 K, inset of Figure 6d. Furthermore, the synthetic procedure reported here allows for stable performance upon cycling and operating at high temperatures. The reproducibility measurements of SnTe‐Bi_2_S_3_ nanocomposites can be found in Figure S20 and S21.

## Conclusion

In summary, we presented a simple, scalable and cheap aqueous synthetic method to produce SnTe‐Bi_2_S_3_ nanocomposites by functionalizing the particle surface with bismuth thiolates. The SnTe nanocomposite structural properties are determined by the bismuth thiolates’ decomposition products during the thermal processing. SnTe‐Bi_2_S_3_ nanocomposites contain smaller SnTe grain domains than bare SnTe, Bi_2_S_3_ nanoprecipitates, and Bi_2_S_3_ rich grain boundary complexions. These features allow for a synergistic optimization of both electrical and thermal transport properties. As a result, the peak and average *z* *T* of SnTe‐Bi_2_S_3_ nanocomposites are improved up to 1.3 at 873 K and ≈0.6 at 300–873 K, respectively. These findings pave a promising and versatile path to tune material structural properties taking advantage of the particles’ surface. By functionalizing the surface with an adequate molecule, unique nanocomposites can be made to improve thermoelectric performance.

## Conflict of interest

The authors declare no conflict of interest.

1

## Supporting information

As a service to our authors and readers, this journal provides supporting information supplied by the authors. Such materials are peer reviewed and may be re‐organized for online delivery, but are not copy‐edited or typeset. Technical support issues arising from supporting information (other than missing files) should be addressed to the authors.

Supporting Information

## Data Availability

The data that support the findings of this study are available from the corresponding author upon reasonable request.
