# Supplementary material for: Surface Functionalization of Surfactant‐Free Particles: A Strategy to Tailor the Properties of Nanocomposites for Enhanced Thermoelectric Performance
Source: Angew Chem Int Ed Engl. 2022 Jul 21;61(35):e202207002. doi: 10.1002/anie.202207002 (PMC9542085; doi:10.1002/anie.202207002)
Supplement: Supplementary file 1 — Supporting Information [file ANIE-61-0-s001.pdf]

Angewandte Chemie
Eine Zeitschrift der Gesellschaft Deutscher Chemiker

Supporting Information

Surface Functionalization of Surfactant-Free Particles: A Strategy to Tailor the Properties of Nanocomposites for Enhanced Thermoelectric Performance

*C. Chang, Y. Liu, S. Ho Lee, M. Chiara Spadaro, K. M. Koskela, T. Kleinhanns, T. Costanzo, J. Arbiol, R. L. Brutchey, M. Ibáñez**

Contents

1. Material processing 3

1.1. Chemicals 3

1.2. SnTe nanoparticle synthesis 3

1.3. Bi_2O_3/Bi_2S_3 molecualr complex preparation 3

1.4. Nanoparticle surface treatment 4

1.5. Bulk nanomaterial consolidation 4

2. Structural and chemical characterization. 5

2.1. X-Ray Diffraction 5

2.2. Scanning Electron Microscopy and Transmission Electron Microscopy 5

2.3. Elemental analysis 5

3. Thermoelectric property measurement 5

3.1. Electrical Properties 5

3.2. Thermal properties 6

3.3. Error estimation 7

3.4. Klemens model 7

4. References 20

1. Material processing

1.1. Chemicals

Tin (II) chloride dihydrate ($SnCl_2 \cdot 2H_2O$, 98%), sodium hydroxide (NaOH, pellets 98%), sodium borohydride ($NaBH_4$, 98%), bismuth oxide (Bi_2O_3, 99.99%), and N-Methylformamide (MFA, 99%) were purchased from Fisher Scientific. Tellurium pieces (Te, 100%), ethylenediamine (en, 99%), 1, 2-ethanedithiol (EDT, ≥95.0%), anhydrous acetone (extra dry), and ethanol (95%) were purchased from Sigma-Aldrich. All chemicals were used as received without further purification. Syntheses were carried out using a standard vacuum/dry argon Schlenk line.

1.2. SnTe nanoparticle (NP) synthesis

Large scale SnTe nanoparticles (NPs) were prepared by reacting sodium stannite (Na_2SnO_2) with sodium hydrogen telluride (NaHTe) in water following a procedure previously reported by Guang Han *et al* with slight modifications.[1]

Firstly, $NaBH_4$ (4.54 g, 120 mmol) was first dissolved in 200 ml deionized water, and then Te powder (5.104 g, 40 mmol) was quickly added into the solution. An Ar flow was introduced to prevent the air. The reaction rate between $NaBH_4$ and Te is very slow and takes ~2 h to react entirely. The suspension was stirred until the solution becomes transparent light purple, indicating the formation of NaHTe. In parallel, NaOH (16 g, 400 mmol) and $SnCl_2 \cdot 2H_2O$ (9.826 g, 40 mmol) were mixed with 200 ml of deionized water. The mixture was stirred at room temperature under Ar flow until the solution became transparent, indicating the formation of Na_2SnO_2. When NaHTe solution is ready, the Na_2SnO_2 solution was heated to its boiling point at *ca.* ~101 ^oC with a condenser to assure reflux. Then the freshly prepared NaHTe solution was rapidly injected. Since NaHTe is sensitive to the air, two 100ml syringes are prepared to inject NaHTe. Upon injection, the solution color changed from transparent to black, indicating the SnTe NP formation. The mixture was heated again to 101 ^oC and maintained at this temperature for 30 min. Then the mixture was cooled to room temperature with cooling water. NPs precipitated quickly when the stirring stopped. The transparent supernatant solution was carefully removed. The remaining crude was transferred into centrifuge tubes for further purification.

The SnTe NPs were washed four times with ethanol and acetone alternatively. At each step, SnTe NPs were dispersed by ethanol/ acetone first and then centrifuged at 8000 rpm for 5 min. At last, the washed NPs were dried under vacuum overnight at room temperature and kept in the glovebox for further use.

1.3. Bi_2O_3/Bi_2S_3 molecualr complex preparation

The Bi_2O_3/Bi_2S_3 molecualr complex preparation method applied in this work was developed by R. L. Brutchey *et al.*[2] The solubilities of Bi_2O_3 and Bi_2S_3 in en+EDT solvent (1:10) is ~15-20 % and ~10 %, respectively. Here, we dissolved 150 mg Bi_2O_3 or 90 mg Bi_2S_3 with 1.1 ml en+EDT solvent (1 ml en, 0.1 ml EDT) in N_2-filled vial. The mixture was sonicated for 1 min to accelerate the dissolving processing until complete dissolution. All the Bi_2O_3 and Bi_2S_3molecular complex were prepared fresh before blending with SnTe NPs in MFA.

1.4. Nanoparticle surface treatment

All surface treatments were performed in an inert atmosphere (N_2). We use 5 ml MFA to disperse 0.75g SnTe in a 20 ml vial. SnTe with different molar amounts of Bi_2O_3 and Bi_2S_3 molecular complex was prepared (For Bi_2O_3 molecualr complex: 0.5% Bi_2O_3: 0.05ml, 1% Bi_2O_3: 0.1 ml, 1.5% Bi_2O_3: 0.16 ml, 2% Bi_2O_3: 0.2 ml, 2.5% Bi_2O_3: 0.25 ml, Bi_2O_3: 0.31 ml; For Bi_2S_3-complex: 1% Bi_2S_3: 0.1 ml, 2% Bi_2S_3: 0.2 ml, 3% Bi_2S_3: 0.31 ml. Then the mixture was vigorously stirred (800 rpm) at room temperature for 24 h. After that, the mixture was washed by acetone for 3 times. Specifically, NPs were dispersed by acetone first and then centrifuged at 7800 rpm for 5 min each time.

Samples for LC/Q-TOF were prepared from fully dissolved solutions of Bi_2O_3 (150 mg/mL, 1:10 (vol/vol) EDT/en) filtered through a 0.45 μm syringe filter and diluted with DMF to nanomolar concentrations. Fully dissolved solutions of Bi_2S_3 (90 mg/mL, 1:10 (vol/vol) EDT/en) were filtered through a 0.45 μm syringe filter and diluted with DMSO to nanomolar concentrations. Both solutions were mixed right before injection into the LC/Q-TOF instrument to avoid possible decomposition products. Samples were direct injected into an Agilent 1290 Infinity ll-6545XT AdvanceBio LC/Q-TOF. UV-vis absorption spectroscopy was performed in a 1-cm path length quartz cuvette placed within a 150 mm integrating sphere on a PerkinElmer Lambda 950 UV-vis-NIR spectrometer with roughly equimolar solutions of Bi_2O_3 (70 mg/mL) diluted in DMF and Bi_2S_3 (80 mg/mL) diluted in DMSO (2 μL ink in 3 mL of either DMF or DMSO). Spectra were collected immediately to avoid any possible absorption from decomposition products.

1.5. Bulk nanomaterial consolidation

As-prepared SnTe-xBi_2O_3/Bi_2S_3 nanocomposites (x=0.5%, 1%, 1.5%, 2%, 2.5%, and 3% Bi_2O_3 molecular complex; x=1%, 2%, and 3% Bi_2S_3 molecular complex) were firstly annealed at 650 °C for 120 min under a slow forming gas (95% N_2 + 5% H_2) flow inside a tube furnace with *ca.* 10 ^{o}C/min heating rate. Afterward, the annealed nano powder was ground with an agate mortar and loaded into a graphite die in the glovebox. The nano powder was then consolidated into pellet (Ø 8.6 mm × h 2 mm) under vacuum in an AGUS PECS Spark Plasma

Sintering (SPS) System-Model SPS 210Sx by applying an axial pressure of 45 MPa at 650 °C for 5 min. All consolidated pellets presented relative densities >98% of the theoretical value.

2. Structural and chemical characterization.

2.1. X-Ray Diffraction (XRD)

X-ray diffraction analyses were carried out on a Bruker AXS D8 ADVANCE powder diffractometer in a Bragg-Brentano geometry with Cu-Kα1 radiation (1.5406 Å). The height of the sample was aligned, and diffraction results were collected from 20° to 80° in 2θ at a rate of 5 °/min. The powder XRD of SnTe NPs with Bi_2O_3 and Bi_2S_3 molecular complex before and after annealing processing were carried out, as shown in **Figure S3**.

2.2. Scanning Electron Microscopy (SEM) and Transmission Electron Microscopy (TEM)

The size and morphology of initial NPs, annealed NPs, and sintered pellets were examined by field-emission SEM on an Auriga Zeiss operated at 5.0 kV. STEM characterization of the Bi_2O_3 molecular complex surface coated SnTe samples has been performed using a JEOL JEM2800 microscope operated at 200 kV with a point to point resolution of 0.14 nm. Besides, STEM-EDS elemental mapping was also performed using a JEOL JEM-2800 microscope at an accelerating voltage of 200 kV with a JEOL silicon drift detector (SDD).

2.3. Elemental analysis

The overall material composition was investigated by using an Oxford energy dispersive X-ray spectrometer (EDX) attached to the Zeiss Auriga SEM at 15.0 kV.

3. Thermoelectric property measurement

3.1. Electrical Properties

Seebeck coefficients were measured by using a static DC method. Electrical resistivity data was obtained by a standard four-probe method. Both the Seebeck coefficient and the electrical resistivity were simultaneously measured in an LSR-3 LINSEIS system from room temperature to 873 K, under a helium atmosphere. The temperature was increased at a rate of 10 K min^{-1}. Data approximately was taken every 30 degrees.

Room-temperature hall charge carrier concentrations (n_H) and mobilities (μ_H) were measured with the Van der Pauw method using a magnetic field of 0.6 T (ezHEMS, NanoMagnetics). Each sample is measured five times to get the average value. Temperature-dependent Hall charge carrier concentrations (n_H) and mobilities (μ_H) from 300 K to 873 K were measured utilizing Hall Effect Analyzer by Linseis Company.

3.2. Thermal properties

An XFA 500 Xenon Flash Apparatus and an LFA 1000 Laser Flash were used to determine the thermal diffusivities (α) of the samples with an estimated error of *ca.* 5 %. The total thermal conductivity was calculated by $\kappa = \alpha C_p \rho$, where α is the thermal diffusivity, C_p is the heat capacity, and ρ is the mass density of the specimen. The temperature-dependent C_p values were derived from reference 3. Notably, we applied the C_p of bare SnTe to estimate C_p in this work becasue the real Bi content in all samples are below 3.5%. In Figure S15, we compared C_p values measured by different groups.

We estimated the lattice thermal conductivity (κ_L) simply by subtracting the electronic thermal conductivity (κ_e) from the measured total thermal conductivity (κ_{tot}):

$$\kappa_L = \kappa_{total} - \kappa_{ele} \tag{S1}$$

The electronic part κ_e is directly proportional to the electrical conductivity σ by the Wiedemann-Franz law;

$$\kappa_{ele} = L\sigma T \tag{S2}$$

An estimation of L can be made using a single parabolic band (SPB) model with acoustic phonon scattering. The calculations based on SPB model results in an L with a deviation of less than 10% as compared with a more rigorous single non-parabolic band and multiple band model calculations. It is well known that the L value is used to estimate the lattice thermal conductivity, which will not change the total thermal conductivity and final zT values. The Lorenz number is given by the formula:

$$L = \left(\frac{k_B}{e}\right)^2 \left(\frac{(r+7/2)F_{r+5/2}(\eta)}{(r+3/2)F_{r+1/2}(\eta)} - \left[\frac{(r+5/2)F_{r+3/2}(\eta)}{(r+3/2)F_{r+1/2}(\eta)}\right]^2 \right) \tag{S3}$$

where k_B is the Boltzmann constant and η represents the reduced Fermi energy, which can be derived from the measured Seebeck coefficients *via* the following equations:

$$S = \pm \frac{k_B}{e} \left(\frac{(r+5/2)F_{r+3/2}(\eta)}{(r+3/2)F_{r+1/2}(\eta)} - \eta \right) \tag{S4}$$

where $F_n(\eta)$ is the n th order Fermi integral:

$$F_\mathrm{n}(\eta)=\int_0^\infty \frac{\chi^\mathrm{n}}{1+e^{\chi-\eta}}\mathrm{d}\chi \tag{S5}$$

$$\eta=\frac{E_\mathrm{f}}{k_\mathrm{B}T} \tag{S6}$$

3.3. Error estimation

Taking the system accuracy and the measurement precision (including the measurement of sizes/distances) into account, we estimate an error of *ca.* 4 % in the measurement of both electrical conductivity and Seebeck coefficient. Combining the uncertainties of electrical conductivity and Seebeck coefficient, so the uncertainty of the power factor is *ca.* 12%. An LFA 1000 Laser Flash were used to determine the thermal diffusivities (α) of the samples with an estimated error of *ca.* 5 %. The density (ρ) error is ~2%. To avoid cluttering the plots, error bars were not included in the figures except zT. Therefore, the combined uncertainty for all measurements involved in zT determination shown in the plot is estimated to be *ca.* 17%.

3.4. Klemens model

At a temperature above the Debye temperature Θ_D, the ratio of the lattice thermal conductivities including point defects and that of parent material can be expressed as the following equation:[3]

$$\frac{\kappa_\mathrm{lat}}{\kappa_\mathrm{lat,\,p}}=\frac{\tan^{-1}U}{U} \tag{S7}$$

where κ_lat and $\kappa_\mathrm{lat,p}$ are the lattice thermal conductivities of the defected and parent materials, respectively. In this work, $\kappa_\mathrm{lat,p}$ = 2.43 W m^{-1} K^{-1}. U is defined as:

$$U=\left(\frac{\pi^2\Theta_D\Omega}{hv_\mathrm{a}^2}\kappa_\mathrm{lat,p}\Gamma\right)^{\frac{1}{2}} \tag{S8}$$

where Θ_D, Ω, h and v_a, represent the Debye temperature, average atom volume, Planck constant and average sound velocity, respectively. Γ, the imperfection scaling parameter is a weighted sum of the mass fluctuation Γ_M and strain field fluctuation Γ_S and can be written as: $\Gamma=\Gamma_\mathrm{M}+\varepsilon\Gamma_\mathrm{S}$, where ε is a phenomenological adjustable parameter related to the Poisson ratio (v_P) and Grüneisen parameter (γ). v_P and γ can be written as:

$$v_{\mathrm{p}} = \frac{1-2\left(v_{\mathrm{s}}/v_{\mathrm{l}}\right)^{2}}{2-2\left(v_{\mathrm{s}}/v_{\mathrm{l}}\right)^{2}} \tag{S9}$$

$$\gamma = \frac{3}{2}\left(\frac{1+v_{\mathrm{p}}}{2-3v_{\mathrm{p}}}\right) \tag{S10}$$

where v_{l} and v_{s} denote longitudinal and shear sound velocities, respectively. Then, we can obtain v_{a} as

$$v_{a} = \left[\frac{1}{3}\left(\frac{1}{v_{\mathrm{l}}^{3}}+\frac{1}{v_{\mathrm{s}}^{3}}\right)\right]^{-\frac{1}{3}} \tag{S11}$$

Setting Bi alloying as an example. Γ is defined as:

$$\Gamma_{\mathrm{Sn_{1-x}Bi_{x}Te}} = \frac{1}{2}\left(\frac{M_{(\mathrm{Sn,Bi})}}{\overline{M}}\right)^{2}\Gamma_{(\mathrm{Sn,\ Bi})} \tag{S12}$$

$$\overline{M} = \frac{1}{2}\left(M_{\mathrm{Sn}}+M_{\mathrm{Bi}}\right) \tag{S13}$$

$$\Gamma_{(\mathrm{Sn,Bi})} = \Gamma_{M(\mathrm{Sn,Bi})}+\varepsilon\Gamma_{S(\mathrm{Sn,Bi})} \tag{S14}$$

$$\Gamma_{M(\mathrm{Sn,Bi})} = x\left(1-x\right)\left(\frac{\Delta M}{M_{(\mathrm{Sn,Bi})}}\right)^{2} \tag{S15}$$

$$\Gamma_{S(\mathrm{Sn,Bi})} = x\left(1-x\right)\left(\frac{\Delta r}{r_{(\mathrm{Sn,Bi})}}\right)^{2} \tag{S16}$$

where $M_{(\mathrm{Sn,Bi})} = \left(1-x\right)M_{\mathrm{Sn}}+xM_{\mathrm{Bi}}$, $\Delta M = M_{\mathrm{Bi}}-M_{\mathrm{Sn}}$ and $r_{(\mathrm{Sn,Bi})} = \left(1-x\right)r_{\mathrm{Sn}}+xr_{\mathrm{Bi}}$, $\Delta r = r_{\mathrm{Bi}}-r_{\mathrm{Sn}}$

Then we get:

$$\Gamma_{\mathrm{Sn_{1-x}Bi_{x}Te}} = \frac{1}{2}x\left(1-x\right)\left(\frac{M_{(\mathrm{Sn,Bi})}}{\overline{M}}\right)^{2}\left[\left(\frac{\Delta M}{M_{(\mathrm{Sn,Bi})}}\right)^{2}+\varepsilon\left(\frac{\Delta r}{r_{(\mathrm{Sn,Bi})}}\right)^{2}\right] \tag{S17}$$

The mass fluctuation Γ_{M} and strain field fluctuation Γ_{S} of S alloying can be obtained using the same method.

Back to the equation S7, we can obtain the theoretical predication κ_{lat} with Klemens model.

4. Figures

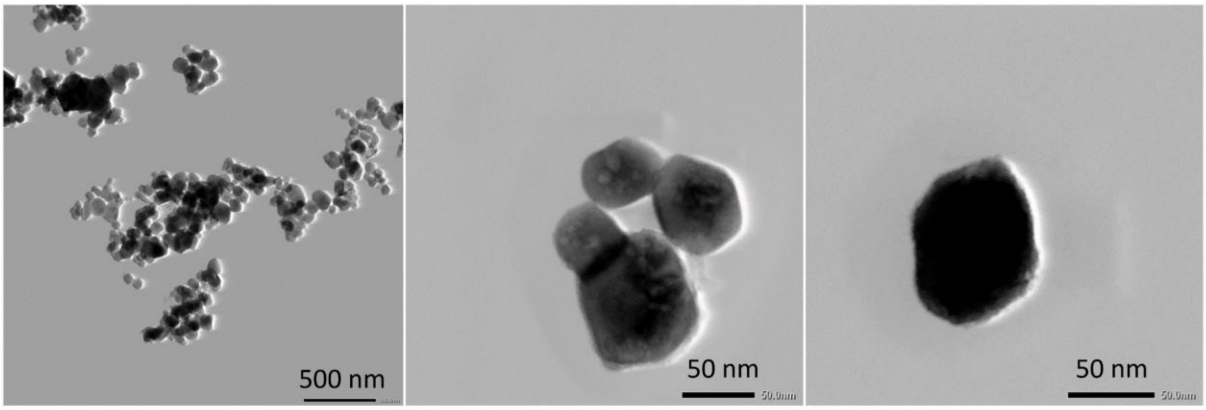

Figure S1. Annular dark-field scanning transmission electron microscope (ADF-STEM) images of SnTe-1.5%Bi_2O_3 molecular complex NPs.

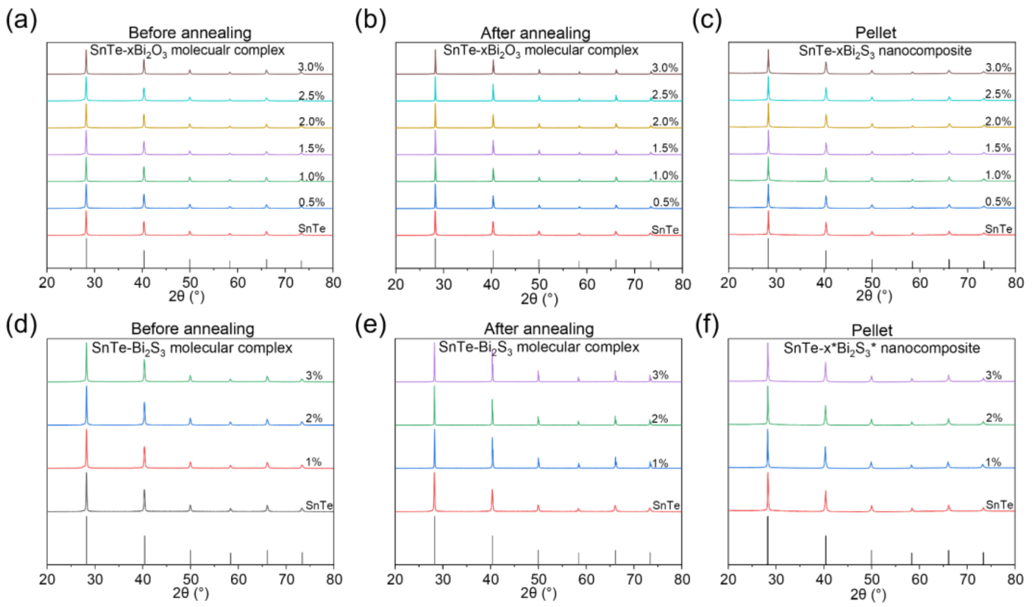

Figure S2. XRD patterns of SnTe-Bi_2O_3 molecular complex NPs (a) before and (b) after annealing processing, and (c) the corresponding SnTe-Bi_2S_3 nanocomposites; XRD patterns of SnTe-Bi_2S_3 molecular complex NPs (d) before and (e) after annealing processing, and (f) the corresponding SnTe-*Bi_2S_3* nanocomposites.

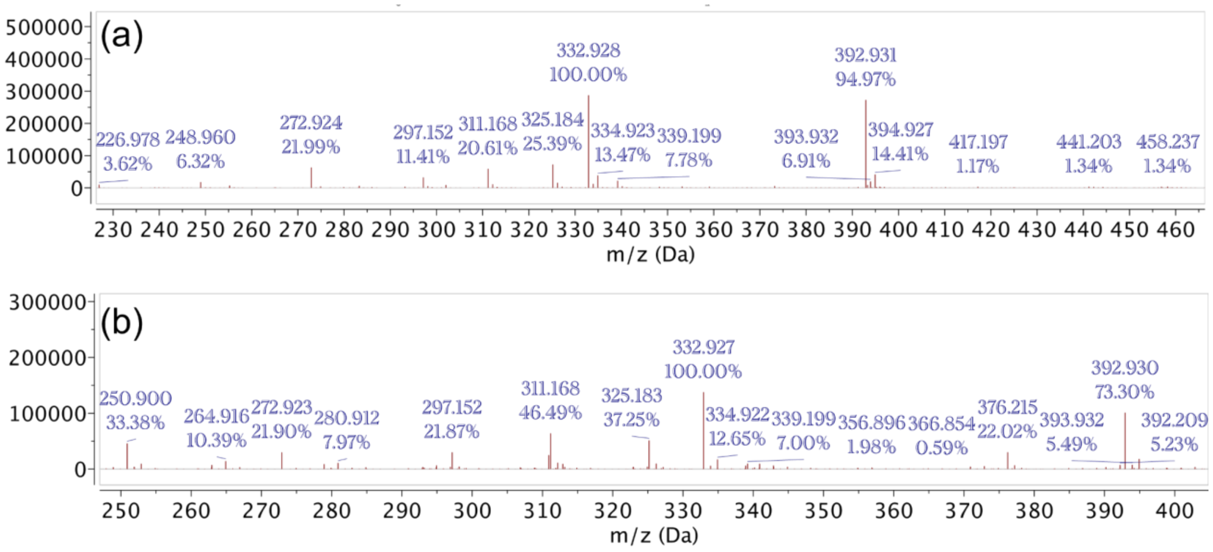

Figure S3. Negative ion mode LC/Q-TOF spectra of direct injected (a) Bi_2O_3 (150 mg/mL, 1:10 (vol/vol) EDT/en) molecular complex diluted in DMF and (b) Bi_2S_3 (90 mg/mL, 1:10 (vol/vol) EDT/en) molecular complex diluted in DMSO.

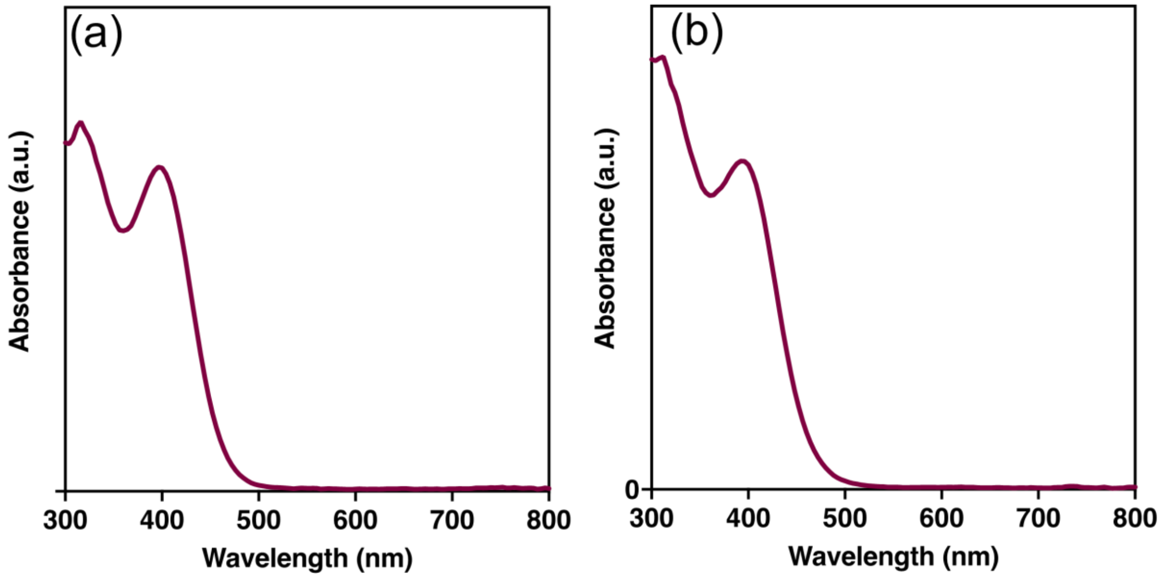

Figure S4. UV-vis absorption spectra of (a) a Bi_2O_3 molecular complex (70 mg/mL, 1:10 (vol/vol) EDT/en) diluted in DMF (2 mL complex in 3 mL DMF). Solvent blank ran with DMF; (b) a Bi_2S_3 molecular complex (80 mg/mL, 1:10 (vol/vol) EDT/en) dissolved in DMF (2 mL complex in 3 mL DMSO). Solvent blank ran with DMSO.

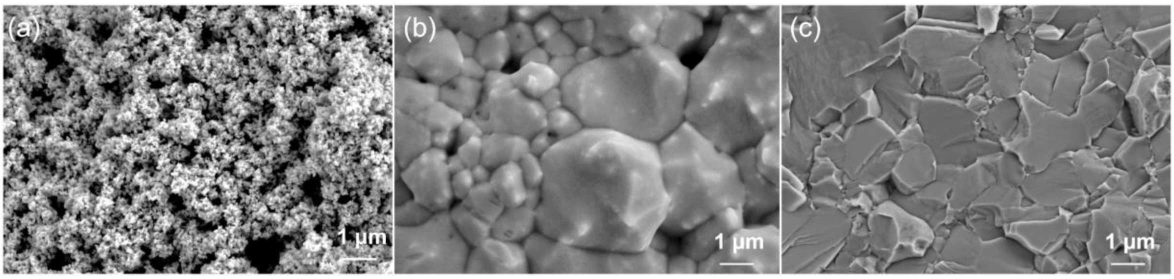

Figure S5. SEM images of SnTe (a) NPs before annealing; (b) NPs after annealing; (c) pellet.

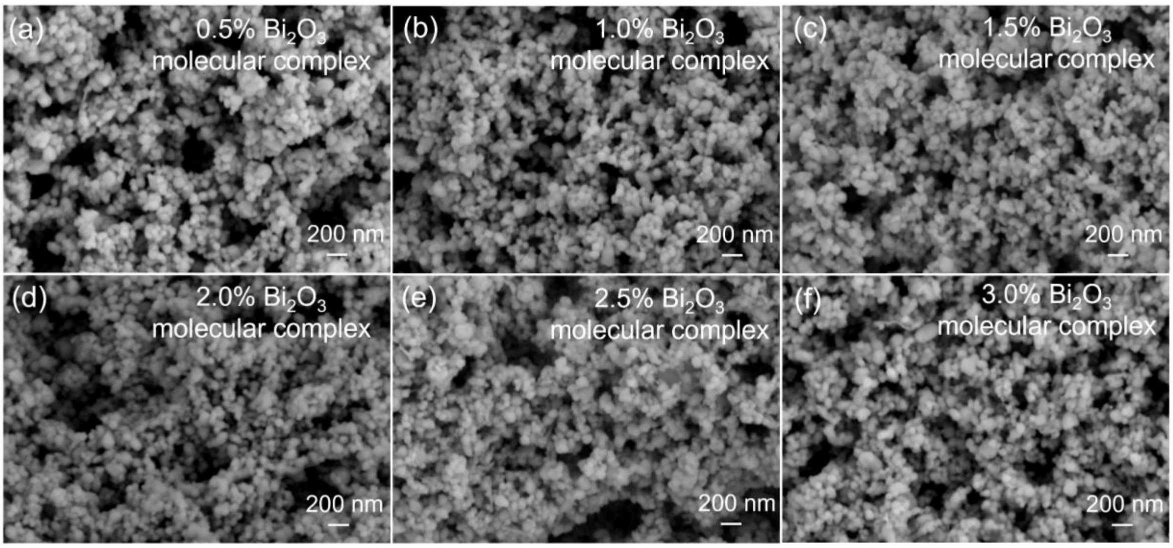

Figure S6. SEM images of the NPs before annealing obtained from (a) SnTe-0.5% Bi_2O_3 molecular complex; (b) SnTe-1% Bi_2O_3 molecular complex; (c) SnTe-1.5% Bi_2O_3 molecular complex; (d) SnTe-2% Bi_2O_3 molecular complex; (e) SnTe-2.5% Bi_2O_3 molecular complex and (f) SnTe-3% Bi_2O_3 molecular complex.

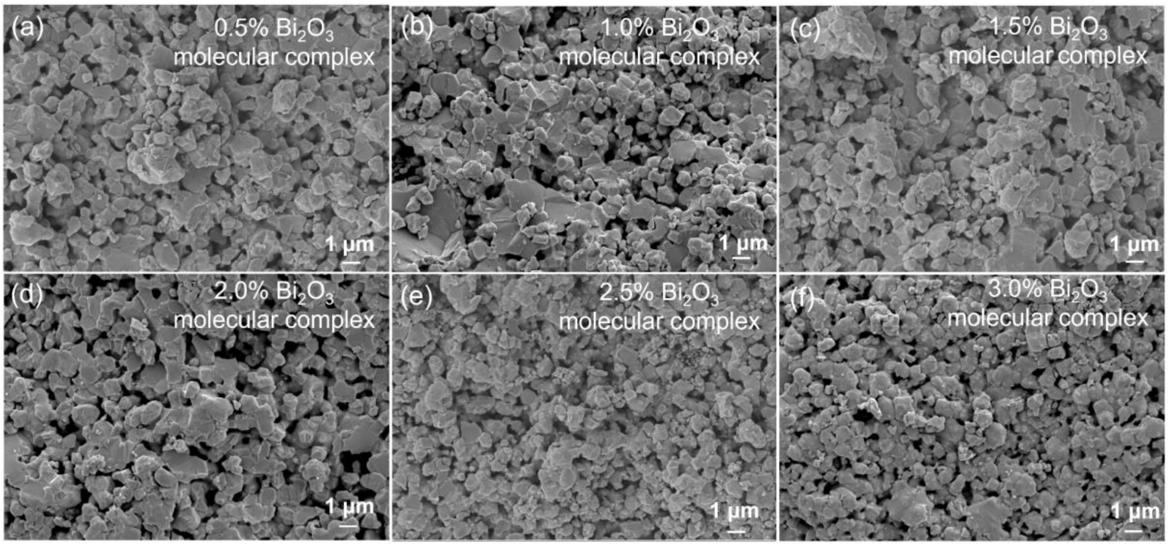

Figure S7. SEM images of the NPs after annealing obtained from (a) SnTe-0.5% Bi_2O_3 molecular complex; (b) SnTe-1% Bi_2O_3 molecular complex; (c) SnTe-1.5% Bi_2O_3 molecular complex; (d) SnTe-2% Bi_2O_3 molecular complex; (e) SnTe-2.5% Bi_2O_3 molecular complex and (f) SnTe-3% Bi_2O_3 molecular complex.

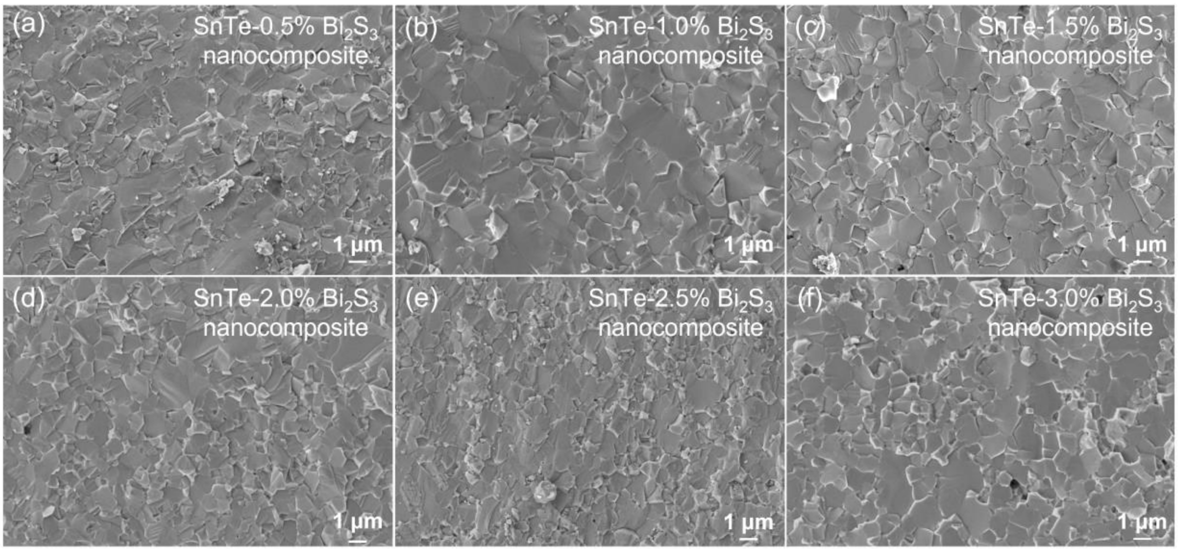

Figure S8. SEM images of the pellets obtained from (a) SnTe-0.5% Bi_2S_3; (b) SnTe-1% Bi_2S_3; (c) SnTe-1.5% Bi_2S_3; (d) SnTe-2% Bi_2S_3; (e) SnTe-2.5% Bi_2S_3 and (f) SnTe-3% Bi_2S_3 nanocomposites produced with Bi_2O_3 molecular complexes as precursor.

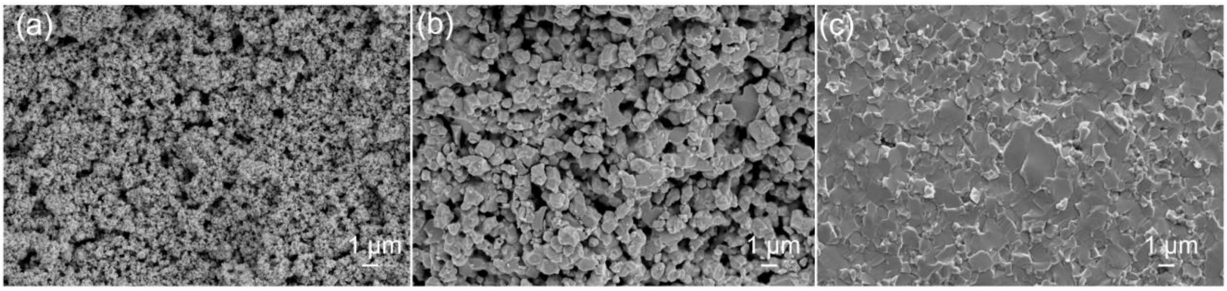

Figure S9. SEM images of SnTe-1% Bi_2S_3 molecular complex (a) NPs before annealing; (b) NPs after annealing; (c) the corresponding pellet.

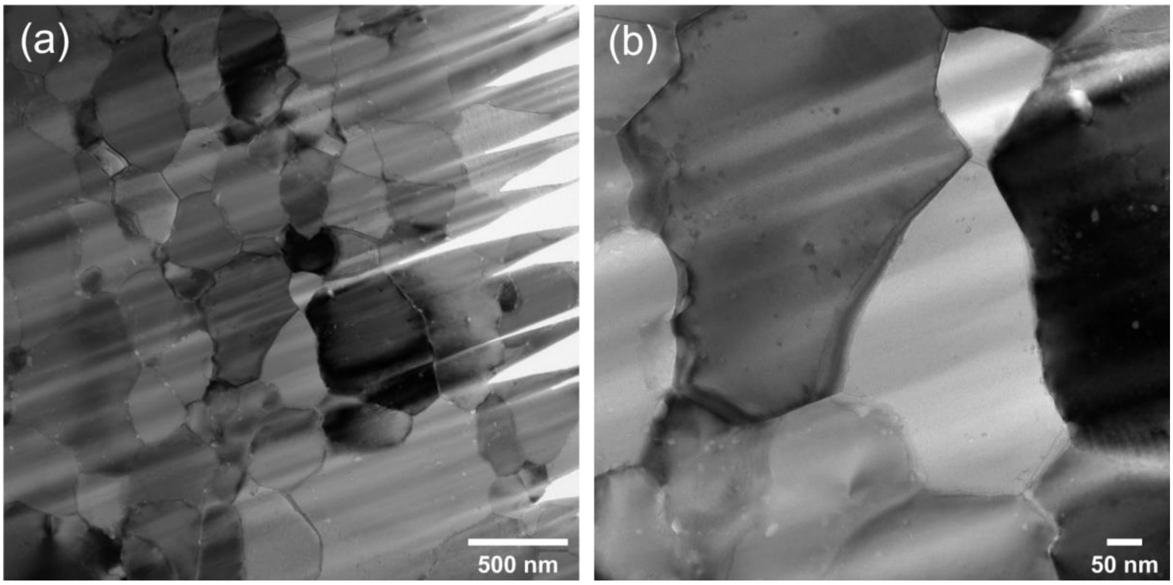

Figure S10. STEM overview in SnTe-Bi_2S_3 nanocomposites produced with 1.5% Bi_2O_3 molecular complexes as precursor.

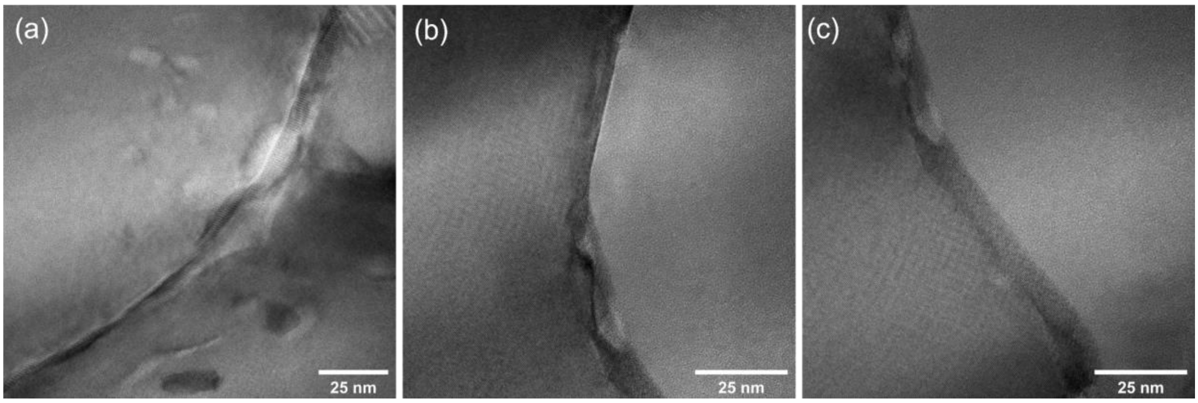

Figure S11. HRTEM of grain boundaries in SnTe-Bi_2S_3 nanocomposites produced with 1.5% Bi_2O_3 molecular complexes as precursor.

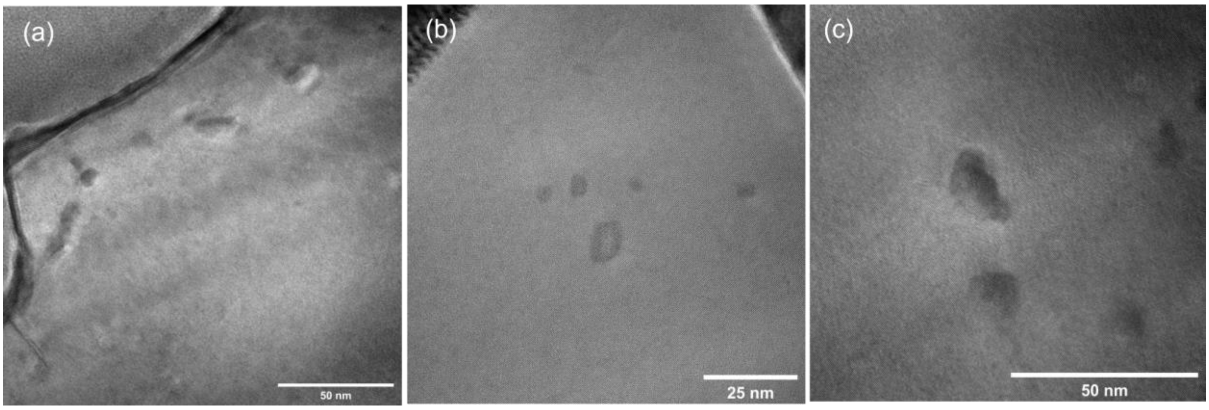

Figure S12. HRTEM of nanoprecipitate in SnTe-Bi_2S_3 nanocomposites produced with 1.5% Bi_2O_3 molecular complexes as precursor.

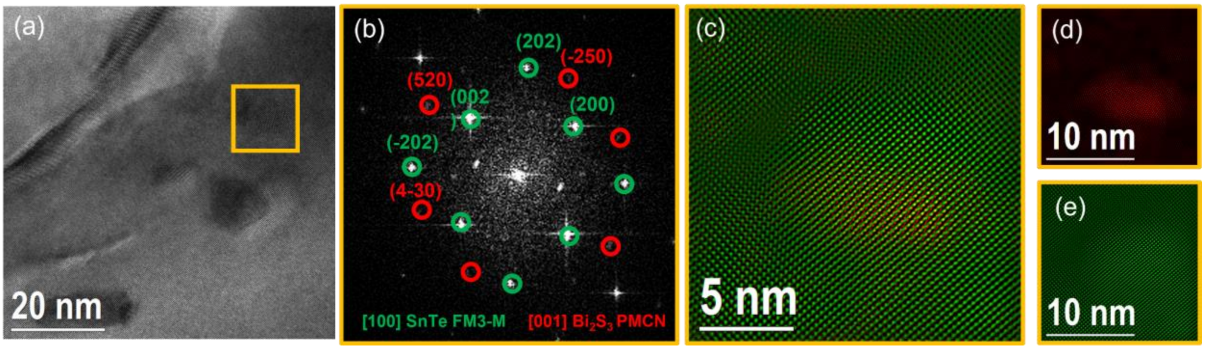

Figure S13. (a) HRTEM of nanoprecipitate in SnTe-Bi_2S_3 nanocomposites produced with 1.5% Bi_2O_3 molecular complexes as precursor. (b) power spectrum of the marked yellow square region. Both SnTe (FM3-M) and Bi_2S_3 (PMCN) are characterized. (c-e) phase-filtered composition maps showing the SnTe phase marked as green and blue and Bi_2S_3 phase marked as red.

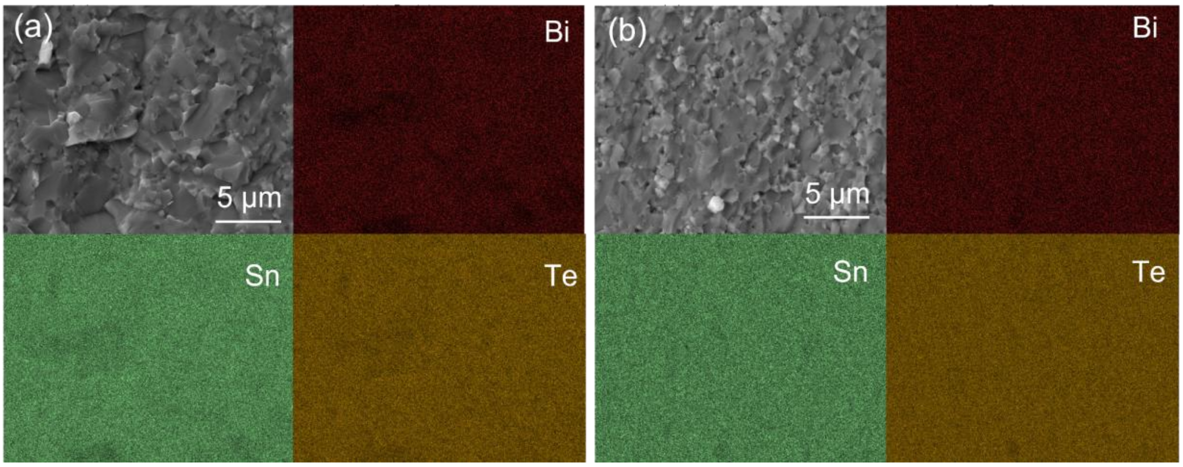

Figure S14. EDS mapping of (a) SnTe-2.5% Bi_2S_3 and (b) SnTe-3.0% Bi_2S_3 nanocomposites produced with Bi_2O_3 molecular complexes as precursor. The EXD resolution is too low to detect the element distribution at the grain boundary.

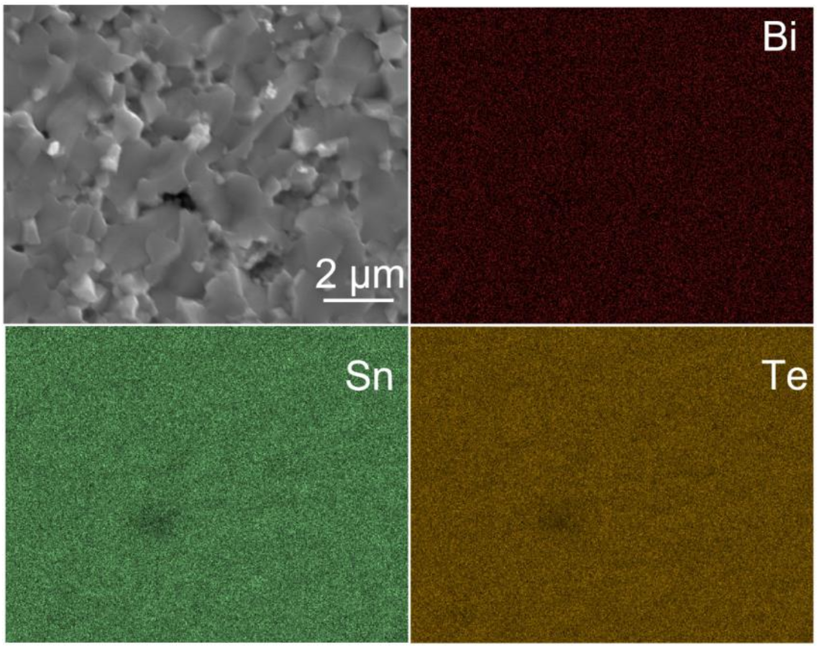

Figure S15. EDS mapping of SnTe-1% *Bi_2S_3* nanocomposite produced with Bi_2S_3 molecular complexes as precursor.

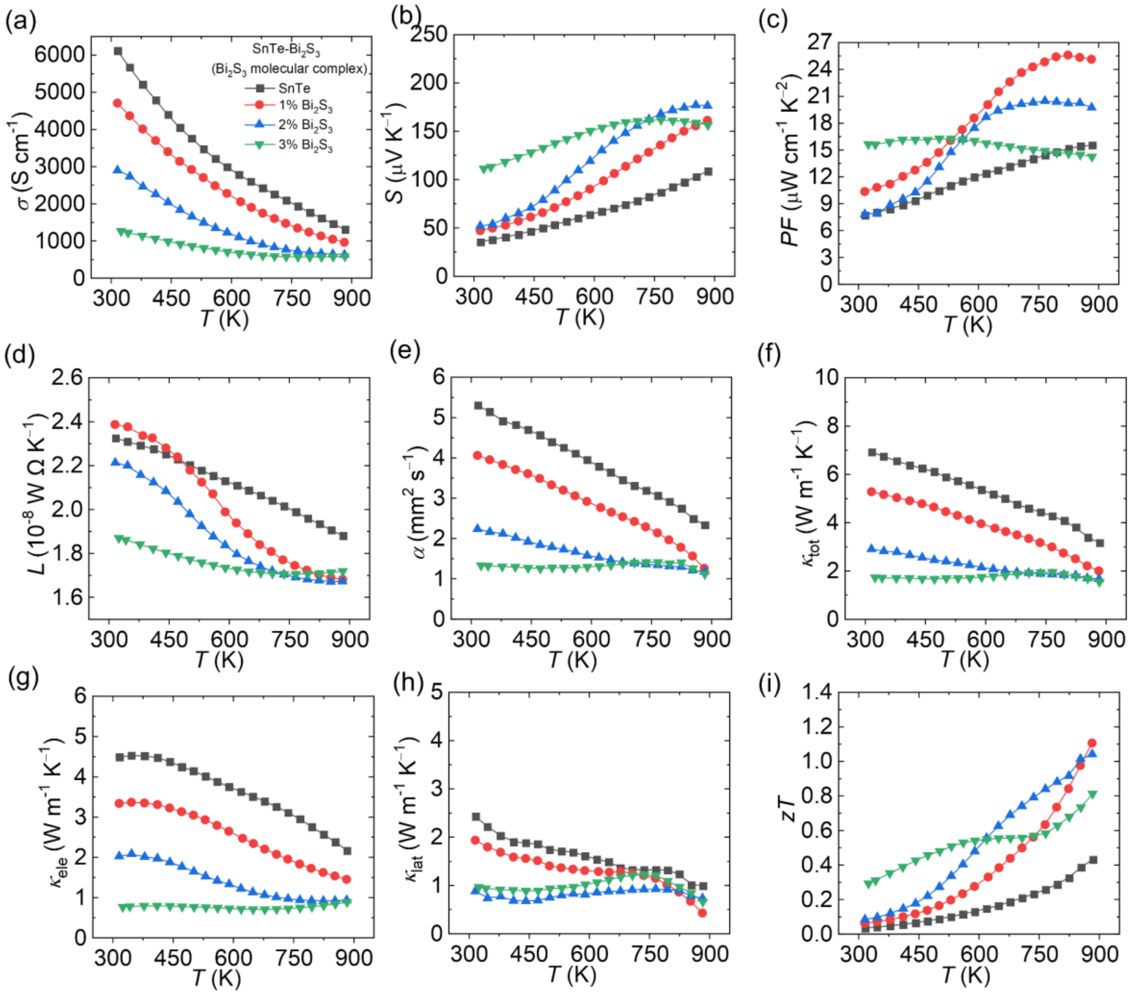

Figure S16. The temperature dependent *TE* properties of SnTe-xBi_2S_3 nanocomposites produced with Bi_2S_3 molecular complexes as precursor (x=0, 1%, 2%, 3%). (a) electrical conductivity; (b) Seebeck coefficient; (c) power factor; (d) Lorenz number; (e) thermal diffusivity; (f) total thermal conductivity; (g) electronic thermal conductivity; (h) lattice thermal conductivity; (f) *zT*.

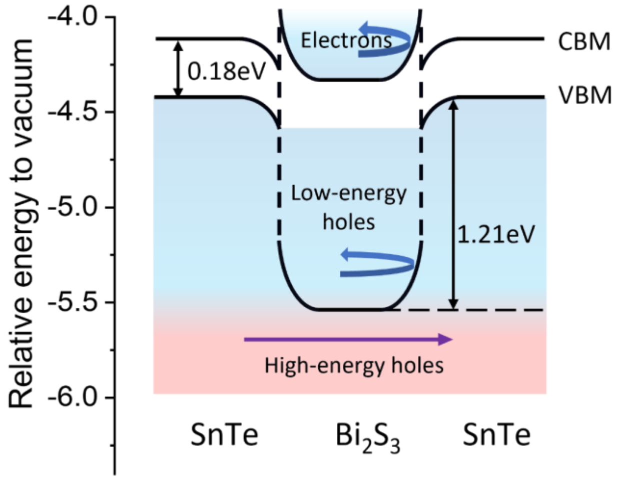

Figure S17. The relative band structure energy of SnTe and Bi_2S_3 to vacuum. The work function of SnTe and Bi_2S_3 are taken from reference [4-5].

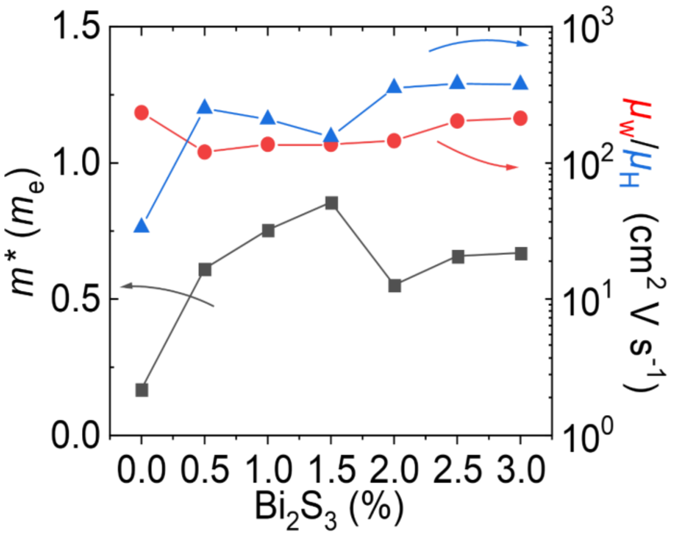

Figure S18. Figure S18. The hall mobility, weighted mobility and effective mass as a function of nominal Bi_2S_3 amount in percentage.

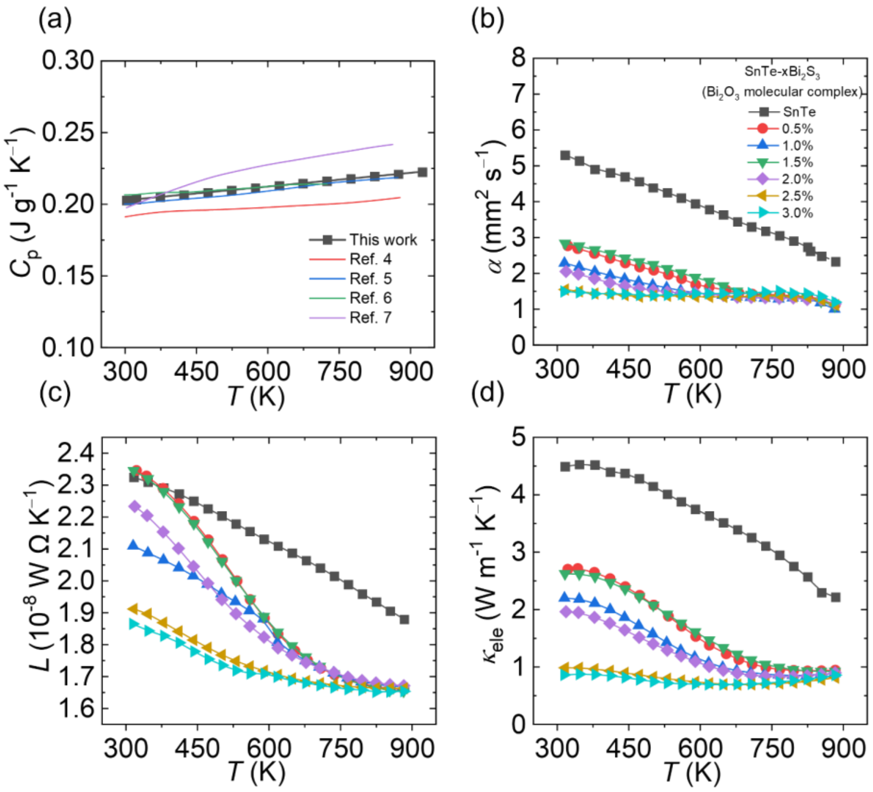

Figure S19. (a) The heat capacity C_p of SnTe as a function of temperature. This figure of C_p values is taken from previous work by Zhao et al.[36] C_p in some other references are listed for comparison;[7-10] The temperature dependent *TE* properties of SnTe-xBi_2S_3 nanocomposites produced with Bi_2O_3 molecular complexes as precursor (x=0, 0.5%, 1%, 1.5%, 2%, 2.5%, 3%). (b) thermal diffusivity; (c) Lorenz number; (d) electronic thermal conductivity.

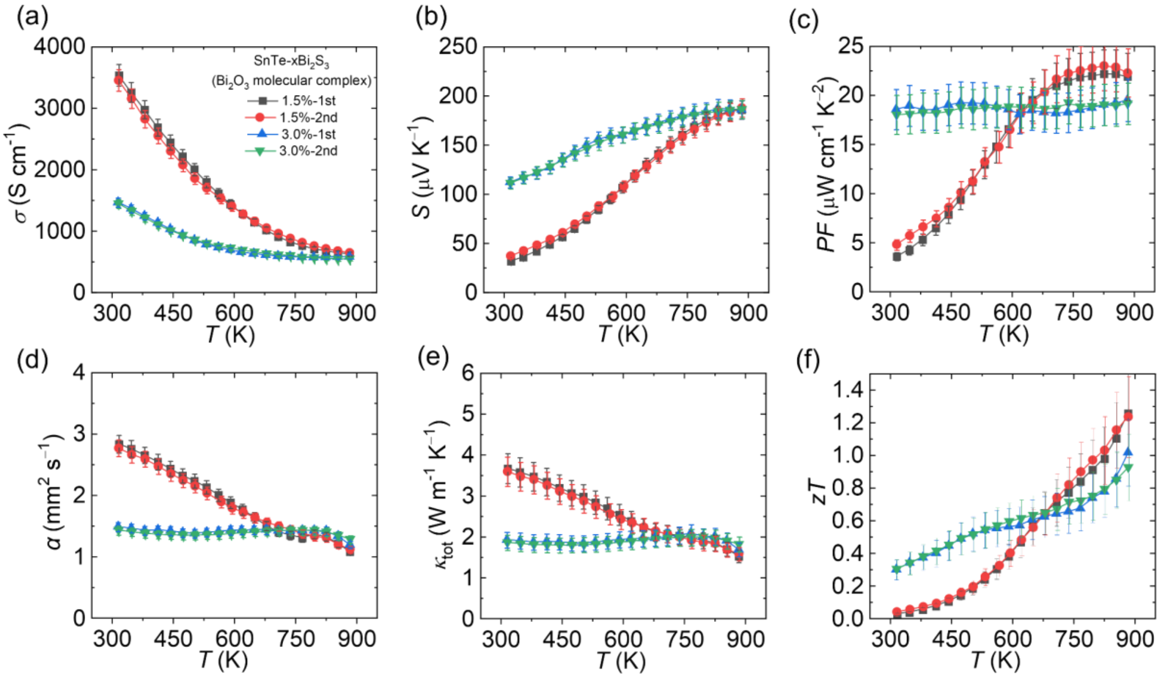

Figure S20. The reproduced TE properties measurement of SnTe-xBi_2S_3 nanocomposites (x=1.5, 3%) produced with Bi_2O_3 molecular complexes as precursor. 1st and 2nd indicates the first and second heating measurement processing (a) electrical conductivity; (b) Seebeck coefficient; (c) power factor; (d) thermal diffusivity; (e) total thermal conductivity; (f) *zT*.

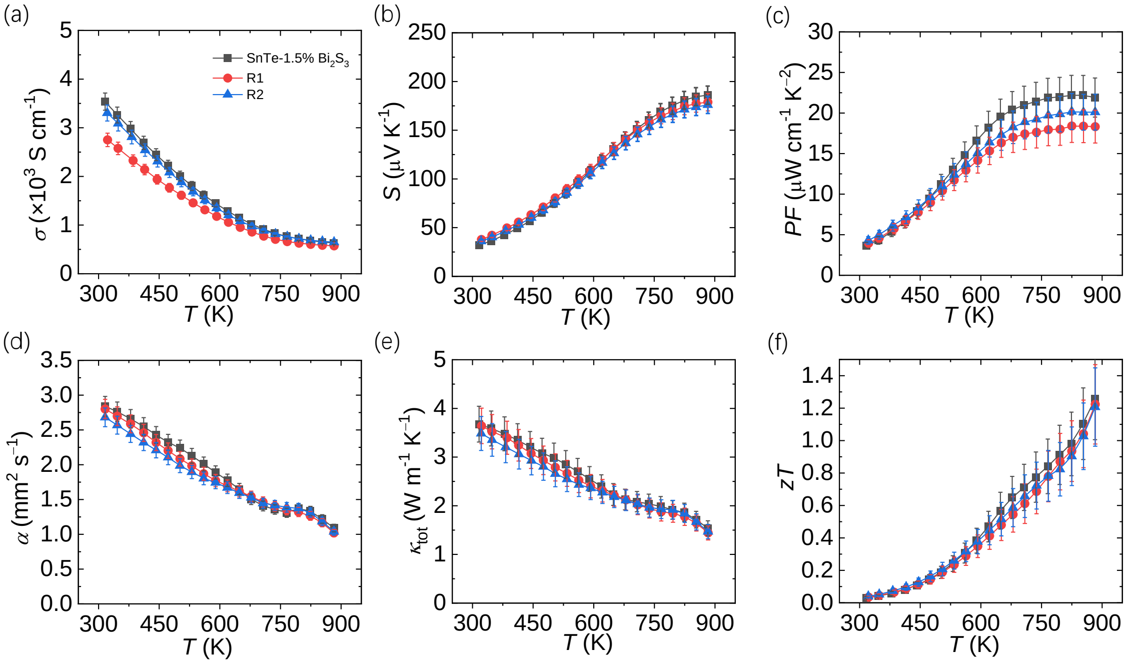

Figure S21. The reproduced TE properties measurement of SnTe-1.5% Bi_2S_3 nanocomposites produced with Bi_2O_3 molecular complexes as precursor. R1 and R2 indicates the SnTe-1.5% Bi_2S_3 nanocomposite synthesized in another two separate batches. (a) electrical conductivity; (b) Seebeck coefficient; (c) power factor; (d) thermal diffusivity; (e) total thermal conductivity; (f) *zT*.

5. Tables

Table S1. Composition of SnTe-xBi_2S_3/*Bi_2S_3* nanocomposites produced with $2O_3$/Bi_2S_3 molecular complexes as precursor obtained from SEM-EDS analysis.

Sample	Sn (atomic %)	Te (atomic %)	Bi (atomic %)	S (atomic %)
SnTe	46.14	53.86	-	-
SnTe-0.5% Bi_2S_3	44.98	53.44	0.67	0.91
SnTe-1.0% Bi_2S_3	44.83	53.05	0.87	1.26
SnTe-1.5% Bi_2S_3	44.98	53.85	0.73	0.43
SnTe-2.0% Bi_2S_3	44.60	53.57	0.94	0.89
SnTe-2.5% Bi_2S_3	43.83	53.78	1.68	0.71
SnTe-3.0% Bi_2S_3	43.42	53.90	1.86	0.81
SnTe-1.0% *Bi_2S_3*	45.59	53.90	0.40	0.11
SnTe-2.0% *Bi_2S_3*	44.48	54.26	1.11	0.15
SnTe-3.0% *Bi_2S_3*	44.10	54.32	1.49	0.09

Table S2. Room temperature Hall carrier concentration and mobility of SnTe-Bi_2S_3/*Bi_2S_3* nanocomposites produced with Bi_2O_3/Bi_2S_3 molecular complexes as precursor.

	n_{Have} (cm^{-3})	μ_{Have} ($cm^2 V s^{-1}$)
SnTe	1.40×10^{21}	33.63
SnTe-0.5% Bi_2S_3	9.37×10^{19}	251.11
SnTe-1.0% Bi_2S_3	6.82×10^{19}	208.90
SnTe-1.5% Bi_2S_3	1.56×10^{20}	155.42
SnTe-2.0% Bi_2S_3	4.47×10^{19}	355.11
SnTe-2.5% Bi_2S_3	2.98×10^{19}	381.435
SnTe-3.0% Bi_2S_3	2.53×10^{19}	376.695
SnTe-1.0% *Bi_2S_3*	4.21×10^{20}	98.21
SnTe-2.0% *Bi_2S_3*	9.70×10^{19}	194.00
SnTe-3.0% *Bi_2S_3*	3.79×10^{19}	256.24

Table S3. Densities of SnTe-xBi_2S_3/*Bi_2S_3* pellets produced with Bi_2O_3/Bi_2S_3 molecular complexes as precursor measured with the Mass-and-volume method.

sample	Density (g/cm^3)
SnTe	6.38
SnTe-0.5% Bi_2S_3	6.37
SnTe-1.0% Bi_2S_3	6.35
SnTe-1.5% Bi_2S_3	6.39
SnTe-2.0% Bi_2S_3	6.38
SnTe-2.5% Bi_2S_3	6.37
SnTe-3.0% Bi_2S_3	6.40
SnTe-1.0% *Bi_2S_3*	6.40
SnTe-2.0% *Bi_2S_3*	6.40
SnTe-3.0% *Bi_2S_3*	6.38

6. References

1. Han, G.; Zhang, R.; Popuri, S. R.; Greer, H. F.; Reece, M. J.; Bos, J.-W. W. G.; Zhou, W.; Knox, A. R.; Gregory, D. H., Large-scale surfactant-free synthesis of p-type SnTe nanoparticles for thermoelectric applications. *Materials* **2017,** *10*, 233.
2. McCarthy, C. L.; Webber, D. H.; Schueller, E. C.; Brutchey, R. L., Solution-phase conversion of bulk metal oxides to metal chalcogenides using a simple thiol-amine solvent mixture. *Angew. Chem., Int. Ed.* **2015,** *54*, 8378-8381.
3. Pei, Y. L.; He, J. Q.; Li, J. F.; Li, F.; Liu, Q. J.; Pan, W.; Barreteau, C.; Berardan, D.; Dragoe, N.; Zhao, L. D., High thermoelectric performance of oxyselenides: intrinsically low thermal conductivity of Ca-doped BiCuSeO. *Npg Asia Mater.* **2013,** *5*, e47.
4. Nishitani, J.; Yu, K. M.; Walukiewicz, W., Charge transfer and mobility enhancement at CdO/SnTe heterointerfaces. *Appl. Phys. Lett.* **2014,** *105* (13), 132103.
5. Han, D.; Du, M.-H.; Dai, C. M.; Sun, D.; Chen, S., Influence of defects and dopants on the photovoltaic performance of Bi_2S_3: first-principles insights. *J. Mater. Chem. A* **2017,** *5* (13), 6200-6210.
6. Zhao, L. D.; Zhang, X.; Wu, H.; Tan, G.; Pei, Y.; Xiao, Y.; Chang, C.; Wu, D.; Chi, H.; Zheng, L.; Gong, S.; Uher, C.; He, J.; Kanatzidis, M. G., Enhanced thermoelectric properties in the counter-doped SnTe system with strained endotaxial SrTe. *J. Am. Chem. Soc.* **2016,** *138*, 2366-2373.
7. Zhang, Q.; Liao, B.; Lan, Y.; Lukas, K.; Liu, W.; Esfarjani, K.; Opeil, C.; Broido, D.; Chen, G.; Ren, Z., High thermoelectric performance by resonant dopant indium in nanostructured SnTe. *Proc. Natl. Acad. Sci. U. S. A.* **2013,** *110*, 13261-13266.
8. Pei, Y.; Zheng, L.; Li, W.; Lin, S.; Chen, Z.; Wang, Y.; Xu, X.; Yu, H.; Chen, Y.; Ge, B., Interstitial point defect scattering contributing to high thermoelectric performance in SnTe. *Adv. Electron. Mater.* **2016,** *2*, 1600019.
9. Banik, A.; Shenoy, U. S.; Anand, S.; Waghmare, U. V.; Biswas, K., Mg alloying in SnTe facilitates valence band convergence and optimizes thermoelectric properties. *Chem. Mater.* **2015,** *27*, 581-587.
10. Zhou, M.; Gibbs, Z. M.; Wang, H.; Han, Y.; Xin, C.; Li, L.; Snyder, G. J., Optimization of thermoelectric efficiency in SnTe: The case for the light band. *Phys. Chem. Chem. Phys.* **2014,** *16*, 20741-20748.
